# Thrombospondin 1 missense alleles induce extracellular matrix protein aggregation and TM dysfunction in congenital glaucoma

**DOI:** 10.1172/JCI156967

**Published:** 2022-12-01

**Authors:** Haojie Fu, Owen M. Siggs, Lachlan S.W. Knight, Sandra E. Staffieri, Jonathan B. Ruddle, Amy E. Birsner, Edward Ryan Collantes, Jamie E. Craig, Janey L. Wiggs, Robert J. D’Amato

**Affiliations:** 1 Vascular Biology Program, Department of Surgery, Boston Children’s Hospital, Boston, Massachusetts, USA.; 2 Department of Ophthalmology, Harvard Medical School, Boston, Massachusetts, USA.; 3 Department of Ophthalmology, Flinders University, Adelaide, South Australia, Australia.; 4 Garvan Institute of Medical Research, Darlinghurst, New South Wales, Australia.; 5 Centre for Eye Research Australia (CERA), Royal Victorian Eye and Ear Hospital, East Melbourne, Victoria, Australia.; 6 Department of Ophthalmology, University of Melbourne, Department of Surgery, Parkville, Victoria, Australia.; 7 Department of Ophthalmology, Royal Children’s Hospital, Parkville, Victoria, Australia.; 8 Department of Ophthalmology, Massachusetts Eye and Ear, Boston, Massachusetts, USA.; 9 Program in Medical and Population Genetics, Broad Institute of MIT and Harvard, Boston, Massachusetts, USA.

**Keywords:** Ophthalmology, Extracellular matrix

## Abstract

Glaucoma is a highly heritable disease that is a leading cause of blindness worldwide. Here, we identified heterozygous thrombospondin 1 (*THBS1*) missense alleles altering p.Arg1034, a highly evolutionarily conserved amino acid, in 3 unrelated and ethnically diverse families affected by congenital glaucoma, a severe form of glaucoma affecting children. *Thbs1^R1034C^*-mutant mice had elevated intraocular pressure (IOP), reduced ocular fluid outflow, and retinal ganglion cell loss. Histology revealed an abundant, abnormal extracellular accumulation of THBS1 with abnormal morphology of juxtacanalicular trabecular meshwork (TM), an ocular tissue critical for aqueous fluid outflow. Functional characterization showed that the *THBS1* missense alleles found in affected individuals destabilized the THBS1 C-terminus, causing protein misfolding and extracellular aggregation. Analysis using a range of amino acid substitutions at position R1034 showed that the extent of aggregation was correlated with the change in protein-folding free energy caused by variations in amino acid structure. Extracellular matrix (ECM) proteins, especially fibronectin, which bind to THBS1, also accumulated within THBS1 deposits. These results show that missense variants altering THBS1 p.Arg1034 can cause elevated IOP through a mechanism involving impaired TM fluid outflow in association with accumulation of aggregated THBS1 in the ECM of juxtacanalicular meshwork with altered morphology.

## Introduction

Glaucoma is a progressive blinding disease that causes loss of retinal ganglion cells (RGCs) and irreversible degeneration of the optic nerve. Elevated intraocular pressure (IOP), currently the only modifiable disease feature, is the target of therapies that can slow disease progression, but these are not curative and cannot restore vision. IOP is determined by the rate of fluid formation (aqueous humor) by the ciliary body and the rate of fluid drainage through trabecular meshwork (TM) and Schlemm’s canal (SC). In addition to the TM/SC pathway, aqueous humor also passes through an alternative or “unconventional” route that includes the ciliary muscle and supraciliary and suprachoroidal spaces (1). In most patients with glaucoma, elevated IOP is the result of reduced fluid outflow, which in the TM/SC pathway is a complex process influenced by ciliary muscle tone, TM cell contractility, and extracellular matrix (ECM) integrity (2).

Overall, glaucoma is a highly heritable disease (3) that can affect people across their lifespan. Early-onset disease (before 40 years of age) is typically the result of Mendelian inheritance, whereas adult-onset glaucoma is inherited as a complex trait (4). Although there are hundreds of genomic loci associated with adult-onset glaucoma and related traits (5–7), there are currently only 10 genes known to cause childhood-onset familial disease. Most of the genes responsible for childhood glaucoma (*CYP1B1*, *LTBP2*, *PITX2*, *PAX6*, *FOXC1*, *TEK*, *ANGPT1*, *CPAMD8*) cause abnormal ocular development (8–11), whereas 2 genes, *MYOC* and *EFEMP1*, are ECM proteins with mutations causing intracellular aggregation in TM cells (12, 13).

Matricellular proteins are secreted glycoproteins that regulate TM cell interaction with the ECM as well as TM remodeling in glaucoma (14, 15) and therefore may be good candidates for glaucoma-causing genes. Thrombospondin 1 (THBS1) is a complex, multifunctional matricellular ECM protein (16) expressed in the juxtacanalicular TM (JCT), the innermost layer of the TM that is critically involved in aqueous fluid outflow (2, 17). Prior studies have shown that in the TM, THBS1 contributes to the regulation of ECM homeostasis and can activate TGF-β (17–19). In human TM cells, cellular stresses related to glaucoma, including exposure to dexamethasone, can increase *THBS1* expression (17), while *Thbs1*-null mice have lower IOP and higher aqueous humor outflow compared with WT mice (20). These studies suggest that THBS1 may contribute to IOP regulation, however, the specific mechanisms involving THBS1 involvement in aqueous fluid outflow are not known.

In this study, we identified 2 *THBS1* missense alleles (R1034C, R1034S) present in affected individuals from 3 unrelated and ethnically diverse families with congenital glaucoma, a severe form of glaucoma affecting children before the age of 3. We show that *Thbs1^R1034C^* mice had elevated IOP, impaired fluid outflow facility, and reduced RGC counts. Furthermore, THBS1 and other ECM proteins were abnormally accumulated in the mutant TM ECM. Functional analyses showed that THBS1 extracellular aggregation was correlated with protein instability caused by variation in amino acid composition at p.Arg1034, including both the cysteine and serine (Ser) changes observed in the affected patients.

## Results

### Identification of THBS1 missense alleles in patients with congenital glaucoma.

**Figure 1:**
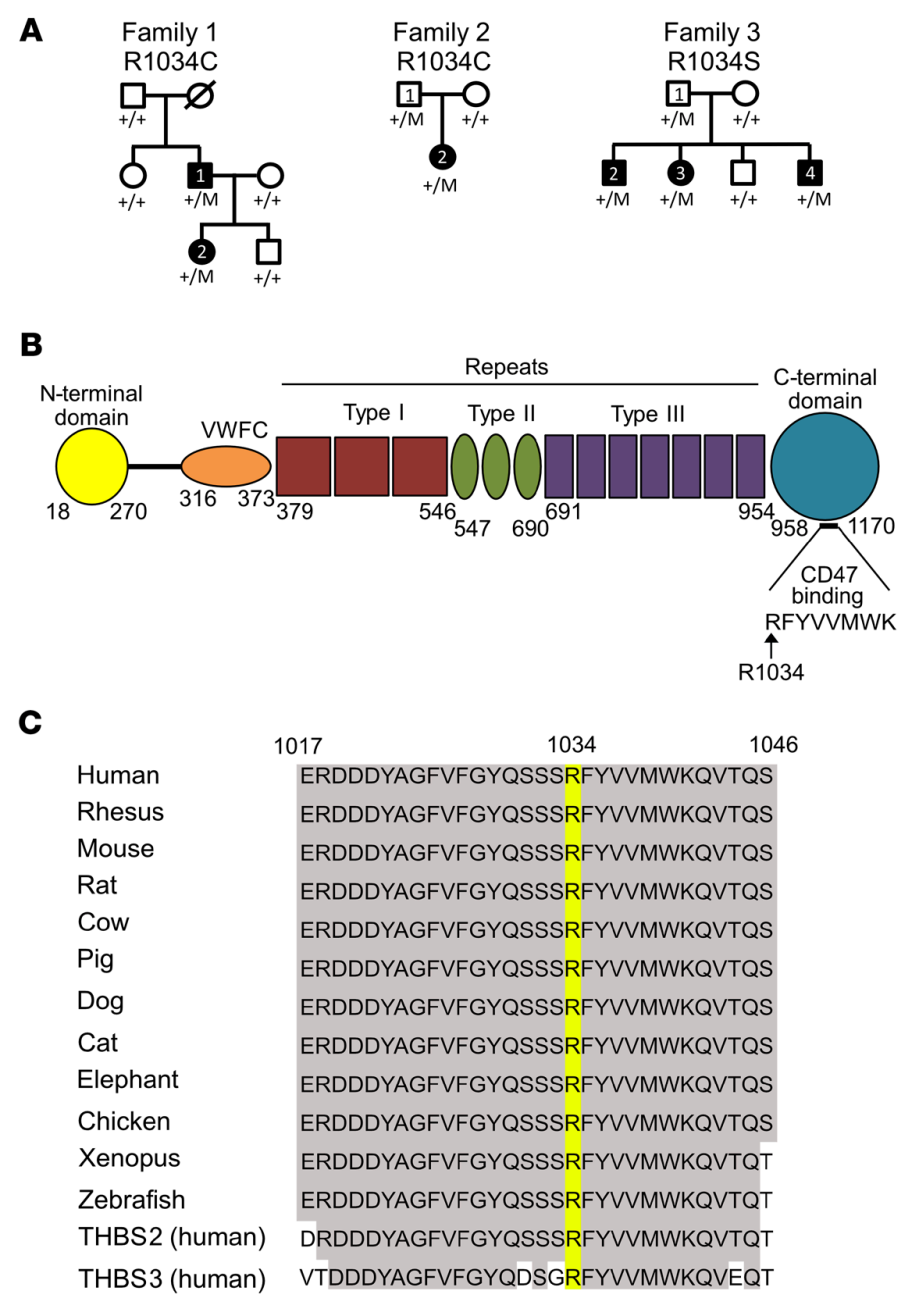
Pedigrees and *THBS1* variants identified in 3 families. (**A**) Pedigrees of 3 families with *THBS1* mutations. Specific mutations in *THBS1* are listed below the family number, with carrier family members annotated as +/M. Affected individuals are indicated by solid black symbols. Note: White symbols do not exclude undiagnosed late-onset disease. (**B**) Schematic representation of THBS1 protein domains and location of R1034 in the C-terminal domain. R1034 is the first amino acid of an 8–amino acid sequence involved with CD47 binding. VWFC, von Willebrand factor type C domain. (**C**) Sequence alignment of THBS1 R1034 showing strong evolutionary conservation.

A heterozygous *THBS1* missense variant (R1034C), apparently segregating in an autosomal-dominant manner, was initially identified by whole-exome sequencing (WES) in a family of European ancestry with congenital glaucoma (family 1, Figure 1). The missense variant was found in both affected family members but in none of the 4 unaffected individuals. We also identified the same R1034C heterozygous variant by exome sequencing in a patient with primary congenital glaucoma who was of mixed European-Indian ancestry and residing in Australia (family 2, Figure 1), and we identified a second missense change at this same residue (R1034S) in 3 siblings with severe primary congenital glaucoma from a family of Sudanese ancestry, who were also residing in Australia (family 3, Figure 1). Neither R1034C nor R1034S was present in any publicly available sequence databases including the Genome Aggregation Database (gnomAD) (gnomad.broadinstitute.org, version 2.1.1), the UK Biobank (genebass.org), and TopMed (https://bravo.sph.umich.edu/freeze8/hg38). Both the father and daughter in family 1 were affected by severe disease and were carriers of R1034C (Table 1). However, at the time of recruitment (at 40 years of age), the father of the severely affected female proband in family 2 did not have a history of early-onset glaucoma, despite being heterozygous for the R1034C variant, suggesting that, like other early-onset glaucoma (*TEK*, *ANGPT1*) (9, 10) or ocular developmental (*MAB21L1*) (21) genes, R1034C may cause a glaucoma phenotype with variable penetrance and/or expressivity. Similarly, the father of the 3 severely affected children in family 3 did not have a history of early-onset glaucoma (age at last follow-up: 38 years), despite being heterozygous for the R1034S variant. We did not identify pathogenic or likely pathogenic variants in other genes known to cause congenital glaucoma, including rare variants (allele frequency <0.001) in *ANGPT1*, *CPAMD8*, *CYP1B1*, *MYOC*, *LTBP2*, *PITX2*, *SVEP1*, and *TEK*. A rare missense variant was identified in *FOXC1* in the proband of family 3 (P17S, GRCh37 chr6:1610729C>T), however, since this variant was present in the population database TopMed, was absent from 1 of the 3 affected children, and was present in an unaffected child and the child’s unaffected mother, it was not considered to be pathogenic, and it did not segregate with a more severe disease phenotype.

We examined single-cell sequence expression data from 10 human anterior segment tissues (22) and found that the highest *THBS1* expression levels were in JCT and the Schwalbe’s cell line, a region that includes TM stem cells (23) (Supplemental Figure 1; supplemental material available online with this article; https://doi.org/10.1172/JCI156967DS1).

### Thbs1^R1034C^-mutant mice exhibit elevated IOP, decreased aqueous humor outflow, and loss of RGCs.

**Figure 2:**
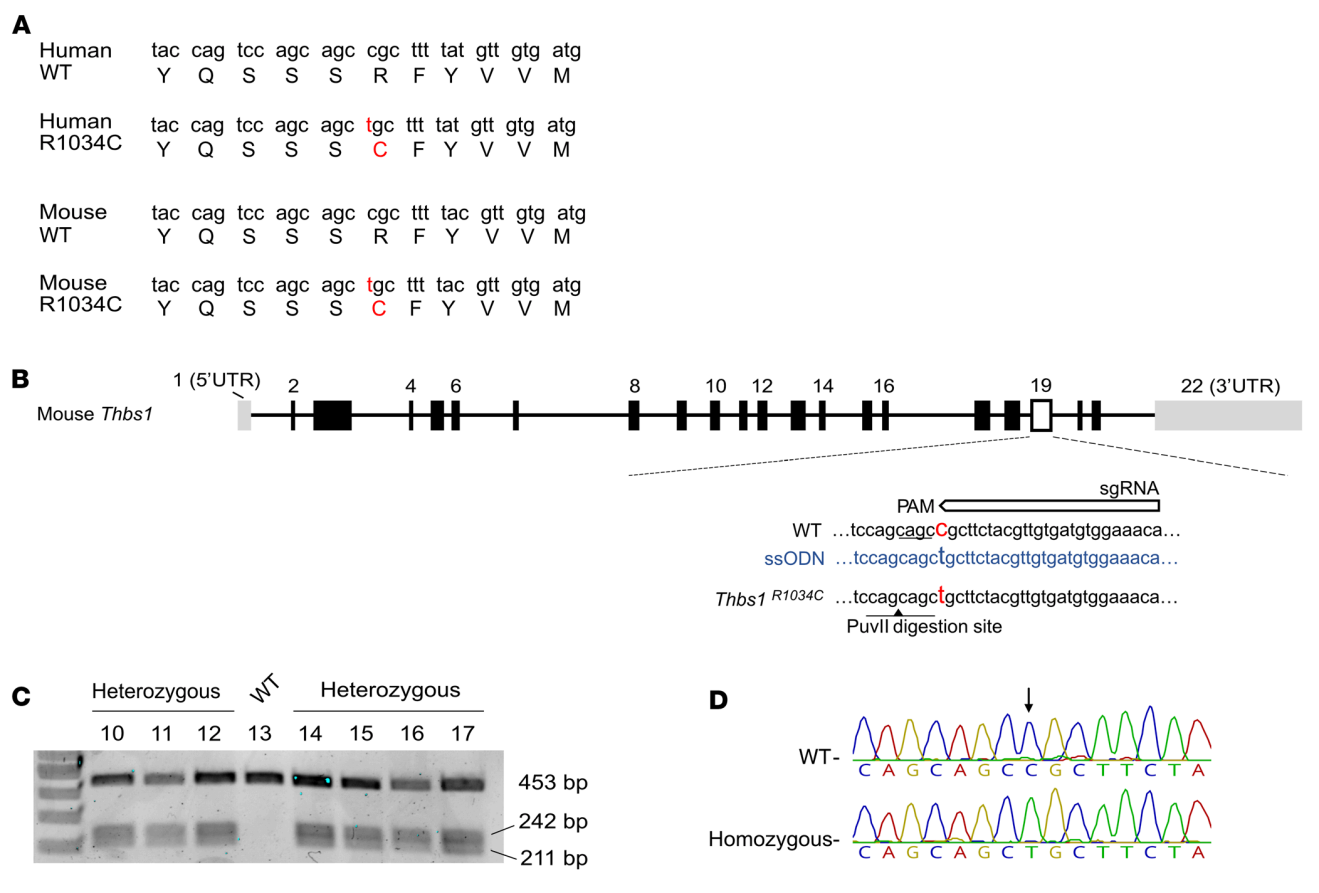
Generation of *Thbs1^R1034C^* mice. (**A**) CRISPR/Cas9 gene editing was used to generate the R1034C mutation in murine *Thbs1*. The R1034 sequence codon is conserved between humans and mice. (**B**) Strategy for single-stranded oligo DNA nucleotide–mediated (ssODN-mediated) knockin with CRISPR/Cas9. Successful mutagenesis generated a PuvII digestion site (black arrowhead) (**C**). Mutants were confirmed by the presence of 242 and 211 bp bands following PCR and PuvII digestion. (**D**) Sanger sequencing confirmed the F1 homozygous versus littermate WT from heterozygous founders in **C**. RNA purified from F1 homozygous mutants also confirmed the point mutation (Supplemental Figure 2).

To determine whether the *THBS1* variants observed in the affected patients were contributing to disease, we first investigated the effects of the R1034C variant using a CRISPR/Cas9 genome-editing approach to generate a mouse line carrying this missense allele (Figure 2, A and B). N1 offspring were born normally and genotyped using PvuII digestion of PCR products (Figure 2C) and Sanger sequencing (Figure 2D). After screening for off-target mutations and back-crossing the missense allele onto WT mice, heterozygous pups were generated as founders for all subsequent experiments. RNA purified from the homozygous mouse mutant confirmed the R1034C variant (Supplemental Figure 2). Both heterozygous and homozygous mice were born normally at a weight comparable to that of WT mice (Supplemental Figure 3). Clinical examination using slit lamp biomicroscopy showed normal anterior segment structures for both heterozygous and homozygous mice (Supplemental Figure 4).

**Figure 3:**
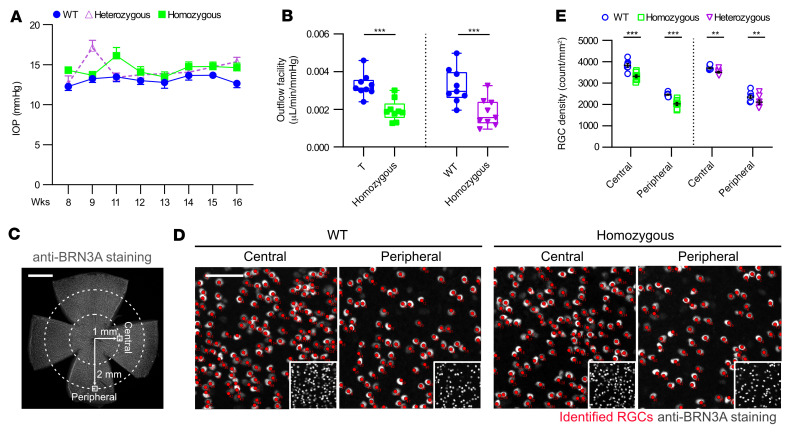
*Thbs1^R1034C^-*mutant mice exhibit elevated IOP, increased outflow resistance, and RGC loss. (**A**) IOP measurements in *Thbs1^R1034C^* homozygous and heterozygous mutant mice. Compared with age-matched WT controls, both homozygous (*P* = 0.0016) and heterozygous (*P* = 0.007) mutant mice showed elevated IOP. Heterozygous, *n* = 14; homozygous, *n* = 18; controls, *n* = 24. Data represent the mean ± SEM. *P* values were determined by repeated-measures 1-way ANOVA followed by Bonferroni’s correction. (**B**) In vivo measurement of aqueous humor outflow facility (reciprocal of outflow resistance) in 4-month-old mice. *Thbs1^R1034C^*-mutant mice showed a significant reduction of outflow facility compared with WT controls. Heterozygous, *n* = 9; heterozygous controls, *n* = 9; homozygous, *n* = 9; homozygous controls, *n* = 9. ***P ≤ 0.01, by 2-tailed Student’s *t* test followed by Bonferroni’s correction. The box boundaries extend from the 25th to the 75th percentiles, the line within the boxes are the mean values, and the whiskers indicate the minimum and maximum values. (**C**) RGC density was determined by BRN3A staining on retinal whole mounts. RGC counts from 6 central and 12 peripheral AOI (AOI = 0.04 mm^2^) were averaged for each eye. Scale bar: 1.0 mm. (**D**) Representative AOI images from **C**. Scale bar: 50 μm; insets, ×0.32. (**E**) Compared with WT mice, there was a significant reduction of RGC density in both the central and peripheral retinas of homozygous (15.9% and 22.2%, respectively, *n* = 8) and heterozygous (5.3% and 10.1%, respectively, *n* = 8) animals. Data represent the mean ± SEM. **P = 0.007 and ****P ≤ 0.0001, by 2-way ANOVA.

Using a noninvasive tonometer, we measured IOP in heterozygous and homozygous mutant mice and compared the values with IOP in age-matched WT mice. IOP measured once per week for 4 months showed consistently higher IOP in both heterozygous and homozygous mutant mice (*P* < 0.001, by 1-way ANOVA) (Figure 3A and Supplemental Figure 5), and IOP continued to be elevated in 7-month-old mice (Supplemental Figure 6). As elevated IOP in glaucoma is correlated with increased resistance in the TM and SC aqueous humor outflow pathways, we next measured outflow resistance in mutant mice. Using a pressure-consistent perfusion system, we found that the outflow facility (C, reciprocal of resistance, μL/min/mmHg) decreased as much as 40.5% in homozygous adult mice (1.95 ± 0.2 vs. 3.29 ± 0.2 μL/min/mmHg, *n* = 9, *P* < 0.001) and 43.3% in heterozygous mice (1.83 ± 0.2 vs. 3.23 ± 0.3 μL/min/mmHg, *n* = 9, *P* = 0.027) (Figure 3B) compared with WT control mice. The increase in trabecular outflow resistance appears to account for the differences in IOP elevation observed between WT and mutant mice, as the alternative uveoscleral outflow pathway was not affected by the THBS1 variant (Supplemental Figure 7). Together, these findings suggest that *Thbs1^R1034C^* mice had impaired aqueous humor outflow through the TM/SC pathway, causing elevated IOP.

As elevated IOP was significantly sustained in both heterozygous and homozygous mutant mice, we assessed the loss of RGCs in retinas from mutant and WT mice using counts of retinal whole mounts (Figure 3, C–E). In homozygous mutant mice, we observed a 15.9% reduction of RGC density in the central retina and a 22.2% reduction in the peripheral retina at 14 months (Figure 3E, *n* = 8, *P* < 0.0001, by 2-way ANOVA). We also observed a reduction in RGC density in heterozygous mice at both the central (5.3%) and peripheral (10.1%) retina (Figure 3E, *n* = 8, ***P = 0.007, by 2-way ANOVA). In addition, we observed a significant increase in degenerating ganglion cell axons in homozygous mice compared with WT mice (143.7% increase, *n* = 8, ***P = 0.0008, by unpaired 2-tailed Student’s *t* test) and, conversely, a decrease in surviving axon bundle density (22.8% reduction, *n* = 8, ****P < 0.0001, by unpaired 2-tailed Student’s *t* test) (Supplemental Figure 8). The percentage of decrease in surviving axons was consistent with the observed percentage decrease of RGCs in the homozygous mice (22.2%). We note that the elevation of IOP, outflow facility, and RGC density were all normal initially in 1-month-old mutant mice (Supplemental Figure 9).

### THBS1 accumulates in the TM in mutant mice.

**Figure 4:**
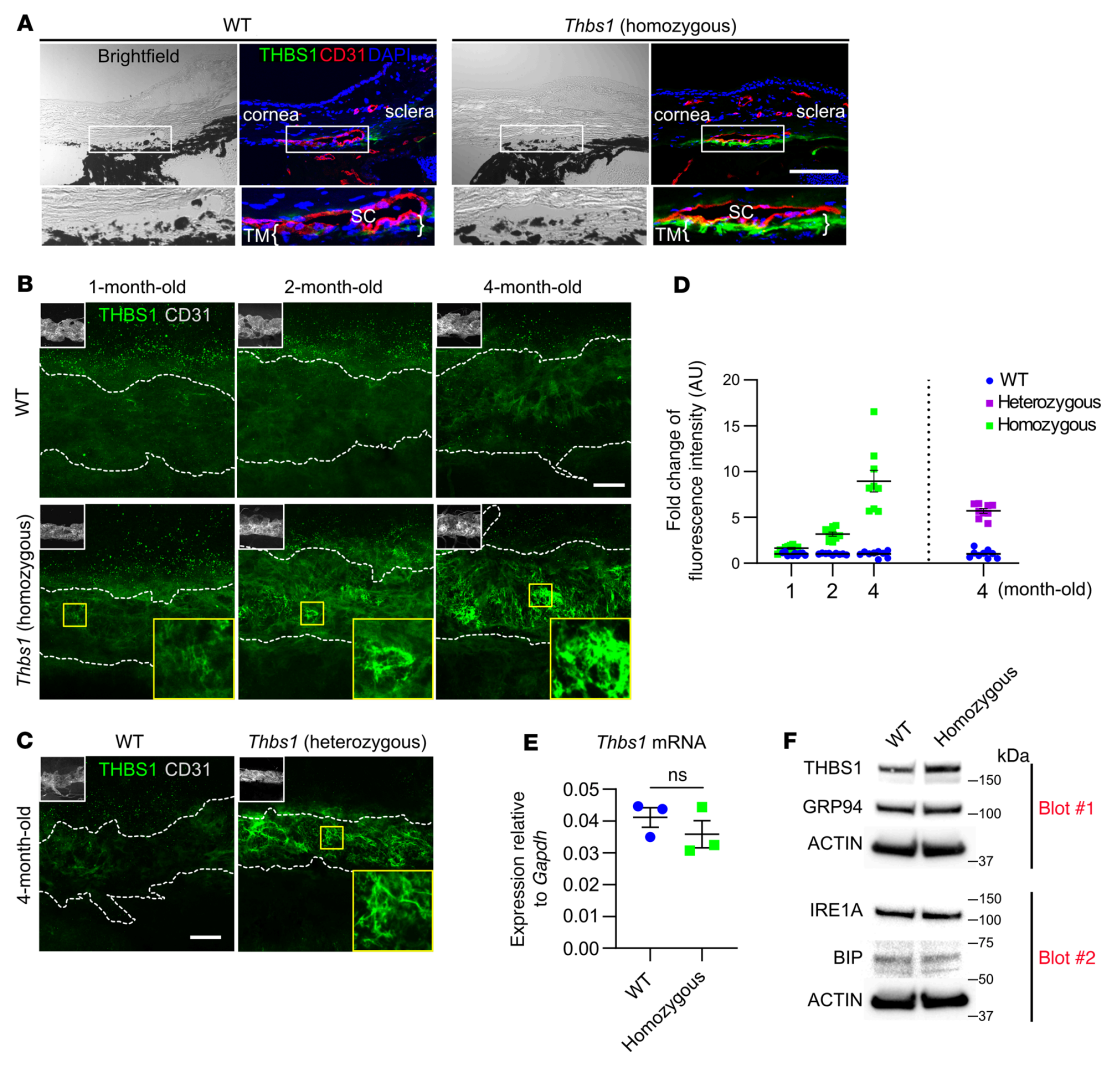
*Thbs1^R1034C^-*mutant mice show accumulation of THBS1 in TM. (**A**) THBS1-stained cryosections of the iridocorneal angle region from 4-month-old *Thbs1^R1034C^* homozygous mice and age-matched controls. TM was localized adjacent to the CD31-stained SC. *Thbs1^R1034C^*-mutant mice showed increased THBS1 protein expression in TM compared with WT controls. Nuclei were stained with DAPI. Scale bar: 100 μm; insets, ×2.4. (**B**) IF staining for THBS1 in corneal whole mounts showing an en face view of protein localization. SC is indicated by dashed lines, as revealed by CD31 staining. Scale bar: 200 μm; insets, ×0.23 (upper left in **B** and **C**). (**C**) Progressive THBS1 protein accumulation in TM was evident in homozygous mice, and a similar pattern of accumulated THBS1 protein was evident in *Thbs^R1034C^* heterozygous mice. Scale bar: 200 μm; insets, ×3.6 (lower right in **B** and **C**). (**D**) Quantification of THBS1 in TM based on fluorescence intensity (100 μm^2^ fields were captured for intensity measurements; *n* = 9 for each time point). AU, arbitrary fluorescence units. Data represent the mean ± SEM. (**E**) *Thbs1^R1034C^* homozygous mice did not show increased *Thbs1* mRNA expression (as determined by unpaired 2-tailed Student’s *t* test). Relative mRNA levels were normalized to *Gapdh*. *n* = 3. Data represent the mean ± SEM. (**F**) Western blot of protein extracts from the corneal limbal ring containing the iridocorneal angle region. Higher levels of THBS1 protein were detected in homozygous *Thbs1^R1034C^* mice, however, there was no evidence of ER stress in the mutant mice at age 16 months of age compared with age-matched controls based on the expression of GRP94, IRE1A, and BIP.

To determine the basis for elevated IOP, we examined the ocular outflow structures in WT and mutant mice using immunohistochemistry. As THBS1 can contribute to the development of vasculature as well as lymphangiogenesis (24), we first considered abnormalities in angiogenesis, lymphangiogenesis, or SC development, as has been recently shown for congenital glaucoma causing mutations in *ANGPT1* and *TEK* (9, 10). Using established assays, we showed that angiogenesis and lymphangiogenesis were normal in *Thbs1^R1034C^* mice and that the SC appeared structurally intact (Supplemental Figure 10). Examination of the TM, however, revealed a substantial increase in THBS1 in heterozygous (Figure 4C) and homozygous mice compared with WT mice (Figure 4, A and B). THBS1 was evident in the youngest mice examined (1 month of age), and protein accumulation increased with age (Figure 4D). The observed increase in THBS1 was not due to increased gene expression (Figure 4E), and the absence of endoplasmic reticulum (ER) stress markers (Figure 4F) suggests that the mutant protein was secreted normally and not retained in the ER. Additionally, an increase in TM cell death was not observed in mutant mice compared with controls (Supplemental Figure 11). THBS1 was absent from the ciliary body (Figure 4A), and THBS1 did not appear to accumulate in other organs, including the colon, liver, lungs, or kidneys of mutant mice (Supplemental Figure 15).

### Mutant THBS1 forms extracellular deposits in TM.

**Figure 5:**
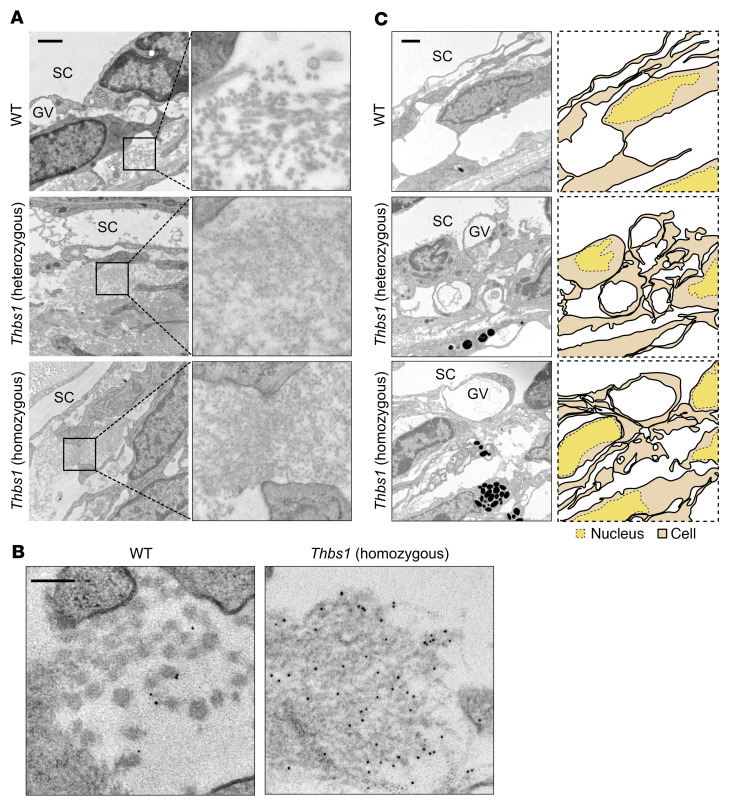
*Thbs1^R1034C^*-mutant mice form aberrant ECM deposition in TM. (**A**) Abnormal ECM deposition was evident in 2-month-old *Thbs1^R1034C^*-mutant mice (homozygous and heterozygous) compared with WT controls, primarily in the JCT adjacent to SC. In mutant mice, a dense fibrogranular material was present in the ECM but was not evident in WT mice, which exhibited organized collagen fibers. Scale bar: 1 μm; enlarged insets, ×5. (**B**) Gold particle labeling was combined with electron microscopy to localize THBS1 protein in TM at the ultrastructural level. Gold particles labeled aggregated THBS1^R1034C^ deposits (small black dots). Scale bars: 0.125 μm. (**C**) Loss of cellularity was evident in the JCT cells of mutant mice, presumably due to the aggregation of THBS1^R1034C^. Scale bar: 1 μm. GV, giant vacuole.

To investigate the basis for impaired outflow facility caused by mutant THBS1, we used transmission electron microscopy (TEM) to visualize aggregated THBS1 in the TM of mutant and WT mice. We identified abnormal extracellular deposits with a distinct fibrogranular structure in both heterozygous and homozygous mutant mice (Figure 5A), mainly in the JCT region adjacent to SC. Additionally, the cellular region of the JCT overlying these deposits was thickened in mutant mice, with an apparent reduction of open spaces (Figure 5C).

Gold particle labeling combined with TEM further confirmed extensive THBS1 deposition in a random distribution (Figure 5) compared with WT mice, in which THBS1 was mainly found adjacent to collagen fibrils (Figure 5B). These results suggest that THBS1^C1034^ formed detrimental extracellular aggregates, unlike other mutant ECM proteins, such as MYOC (25) and EFEMP1 (13), that form intracellular aggregates causing early-onset glaucoma.

### THBS1^C1034^ C-terminal misfolding promotes ECM deposition.

**Figure 6:**
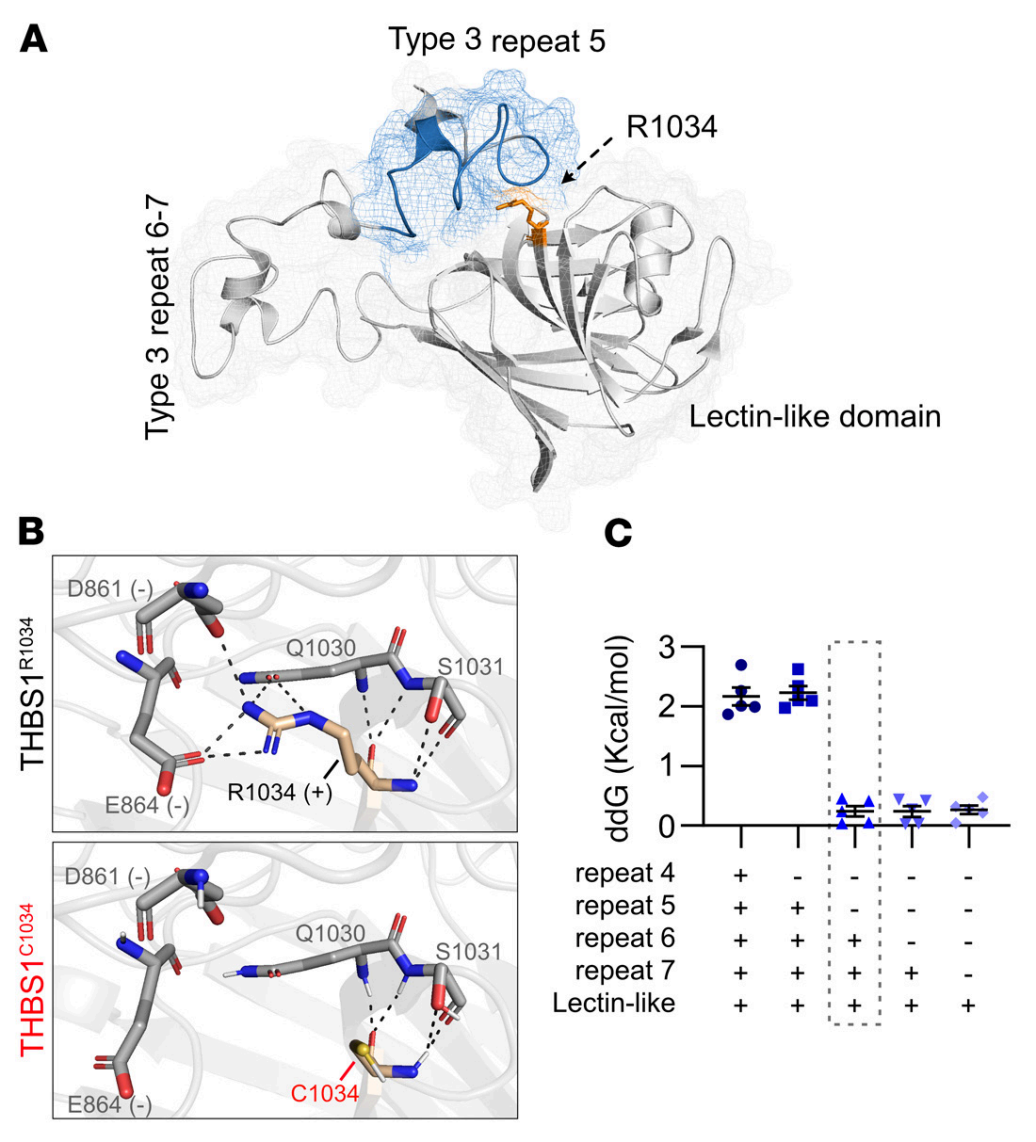
R1034C substitution destabilizes the C-terminal of THBS1. (**A**) The C-terminal of human THBS1 consists of type 3 repeats 4–7 and a lectin-like domain. Arg1034 (orange) in the lectin-like domain interacts with repeat 5 (blue) by intramolecular forces, such as electrostatic and hydrogen bonds. (**B**) Cysteine, however, cannot sustain these interactions due to a shorter and uncharged side chain. (**C**) As a result, the R1034C substitution significantly destabilizes the C-terminal structure (mean ddGB = 2.17 kcal/mol). Of note, the destabilizing effect is dramatically eliminated upon the exclusion of repeat 5 from the C-terminal, indicating the important role of repeat 5 and Arg1034 in protein structure stabilization. ddGB was derived from 5 independent protein stability analyses on FoldX. Data represent the mean ± SEM.

R1034 is in the THBS1 C-terminal domain (Figure 1) and is highly evolutionarily conserved. The C-terminal crystal structure suggests that R1034 is conformationally adjacent to type 3 repeat 5, which has been reported to extensively interact with the C-terminal lectin-like domain (Figure 6A) (26). With its long, positively charged side chain, R1034 forms a hydrogen bond with D861/E864 in repeat 5 (Figure 6B). We hypothesized that the change from arginine to cysteine at position 1034 could disrupt the interaction with the type 3 repeat and expose the buried hydrophobic residues between repeats 6 and 7, resulting in protein aggregation. To assess this hypothesis in silico, we simulated the mutation in an empirical force field–based program, FoldX (27), using the C-terminal fragment structure (1UX6). As expected, R1034C significantly destabilized the THBS1 C-terminal structure by generating 2.17 kcal/mol extra free unfolding energy, ΔΔGibbs (ddG) (>0.5 was significant). Moreover, the ddG was significantly reduced when the repeat 5 was removed from the structure (ddG dropped to 0.24 kcal/mol, Figure 6C), suggesting that unfolding may have occurred between repeat 5 and position 1034, where the amino acid side chain could have an important role in determining the folding state. The structural modeling overall suggested that R1034C may disrupt normal conformation in the C-terminal, thereby promoting protein deposition.

**Figure 7:**
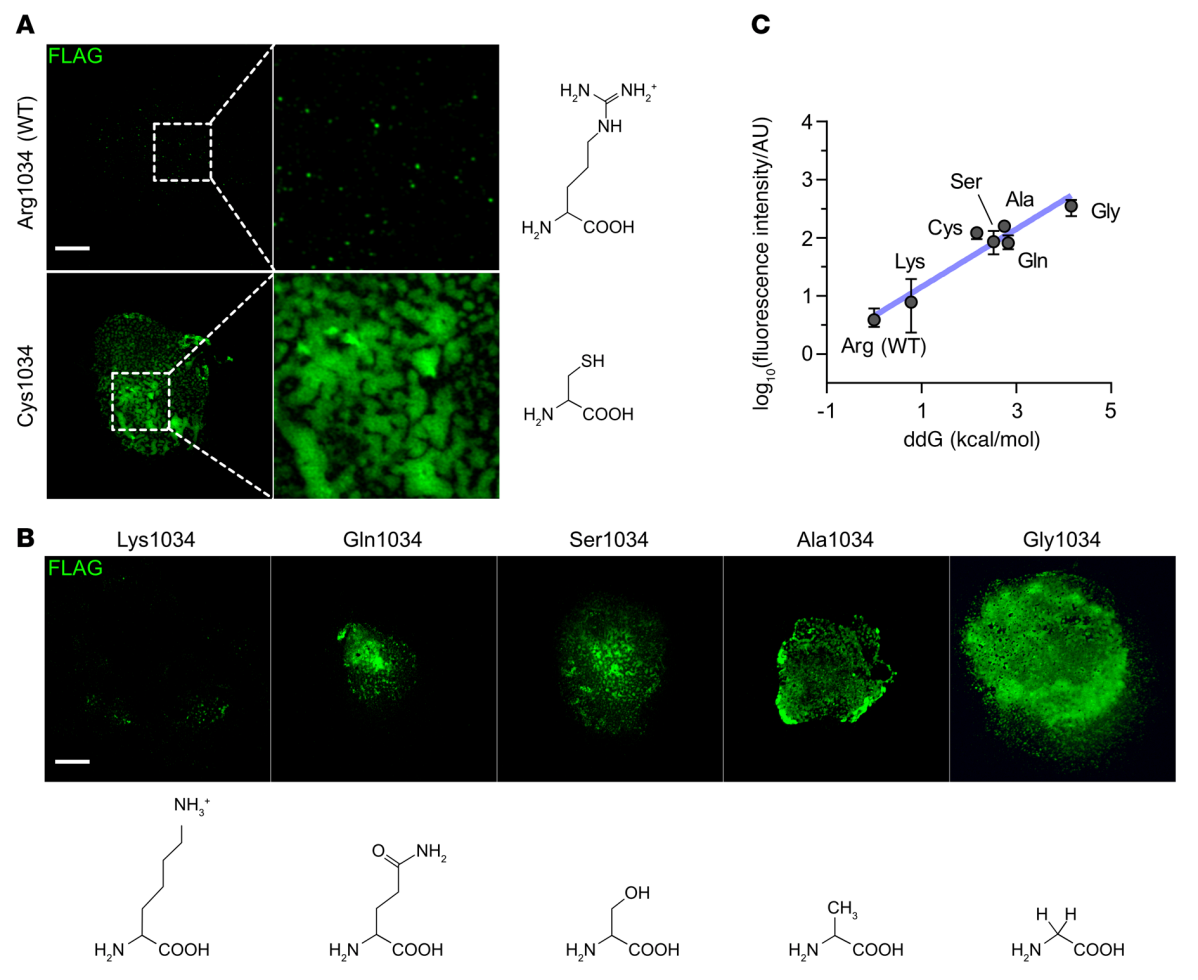
Correlation of predicted protein instability with ECM deposition. (**A**) The propensity for THBS1^R1034C^ aggregation in the ECM was further assessed through ectopic expression of FLAG-tagged THBS1 in COS-7 cells. The absence of DAPI (nuclear acid) and F-actin (cytoskeleton) staining indicated the successful removal of cellular components. THBS1 extracellular deposition was evaluated by anti-FLAG IF staining. Consistent with the in vivo findings, there was greater accumulation of mutant THBS1^C1034^ in ECM than of THBS1^R1034^ (WT). Scale bar: 10 μm; enlarged insets, ×4. (**B**) Plasmids with Arg1034 mutated to Lys, Gln, Ser, Ala, and Gly were transfected into COS-7 cells. ECM deposition was lowest in Lys1034 and greatest in Gly1034, with Gln, Ser, and Ala showing intermediate effects. Scale bar: 10 μm. (**C**) THBS1 ECM deposition strongly correlated with the change in protein stability (*R^2^* = 0.86, *P* < 0.0001, by linear regression), suggesting that Arg1034 had an essential role in reducing abnormal THBS1 ECM deposition. The change in protein stability (ddGB) was derived from 5 analyses in FoldX. Protein deposition was quantified on the basis of fluorescence intensity of anti-FLAG immunostaining from 3 independent experiments. The mean values of ddGB and fluorescence intensity were used for regression analysis. Error bar indicates the SEM.

To further investigate the THBS1^C1034^ extracellular deposits, we generated an equivalent mutation (R1034C) in a FLAG-tagged *Thbs1* cDNA ORF plasmid. WT and mutant plasmids were transfected into COS-7 cells, and THBS1 extracellular deposition was quantified using immunostaining for the FLAG tag. Consistent with the in vivo findings, we observed that extracellular accumulation of mutant THBS1 was increased compared with WT protein levels (Figure 7A, 31-fold change in fluorescence intensity). Qualitatively, the mutant protein formed large aggregates, whereas the WT THBS1 formed small punctate patches, consistent with other studies (26, 28).

To assess the contribution of protein misfolding to abnormal ECM deposition, we created a series of THBS1 R1034-mutant plasmids and tested the aggregation propensity in COS-7 cells after transfection. To examine a range of effects, R1034 was changed to lysine (Lys), Ser, alanine (Ala), glutamine (Gln), and glycine (Gly) with misfolding ddGs of 0.78 kcal/mol, 2.52 kcal/mol, 2.75 kcal/mol, 2.83 kcal/mol, and 4.16 kcal/mol, respectively. Mutagenesis, transfection, and quantification for THBS1 deposition were conducted as described above. Interestingly, we found that the amount of deposition was strongly correlated with the ddG of the mutation (Figure 7C, *R^2^* = 0.86). Moreover, the increase in THBS1 deposition was correlated with a reduction in side chain size or charge (Figure 7B). For example, there was 45-fold less deposition of THBS1^K1034^ than of THBS1^G1034^ (Figure 7C), as lysine is the most similar to the WT arginine, including positive charge and a relatively long side chain, which appear to be important features necessary for maintaining proper conformation. In contrast, glycine has 2 hydrogen atoms in its side chain, rendering more detachment of the repeat 5 from the lectin-like domain and more exposure of hydrophobic residues. Importantly, serine, the amino acid found at R1034 in family 3, had misfolding effects similar to those observed for the cysteine found in the other patients with congenital glaucoma. Collectively, those findings support the hypothesis that substitution of R1034 with cysteine or serine promotes THBS1 deposition in ECM by disrupting C-terminal folding.

### THBS1^C1034^ deposition recruits interstitial ECM proteins.

**Figure 8:**
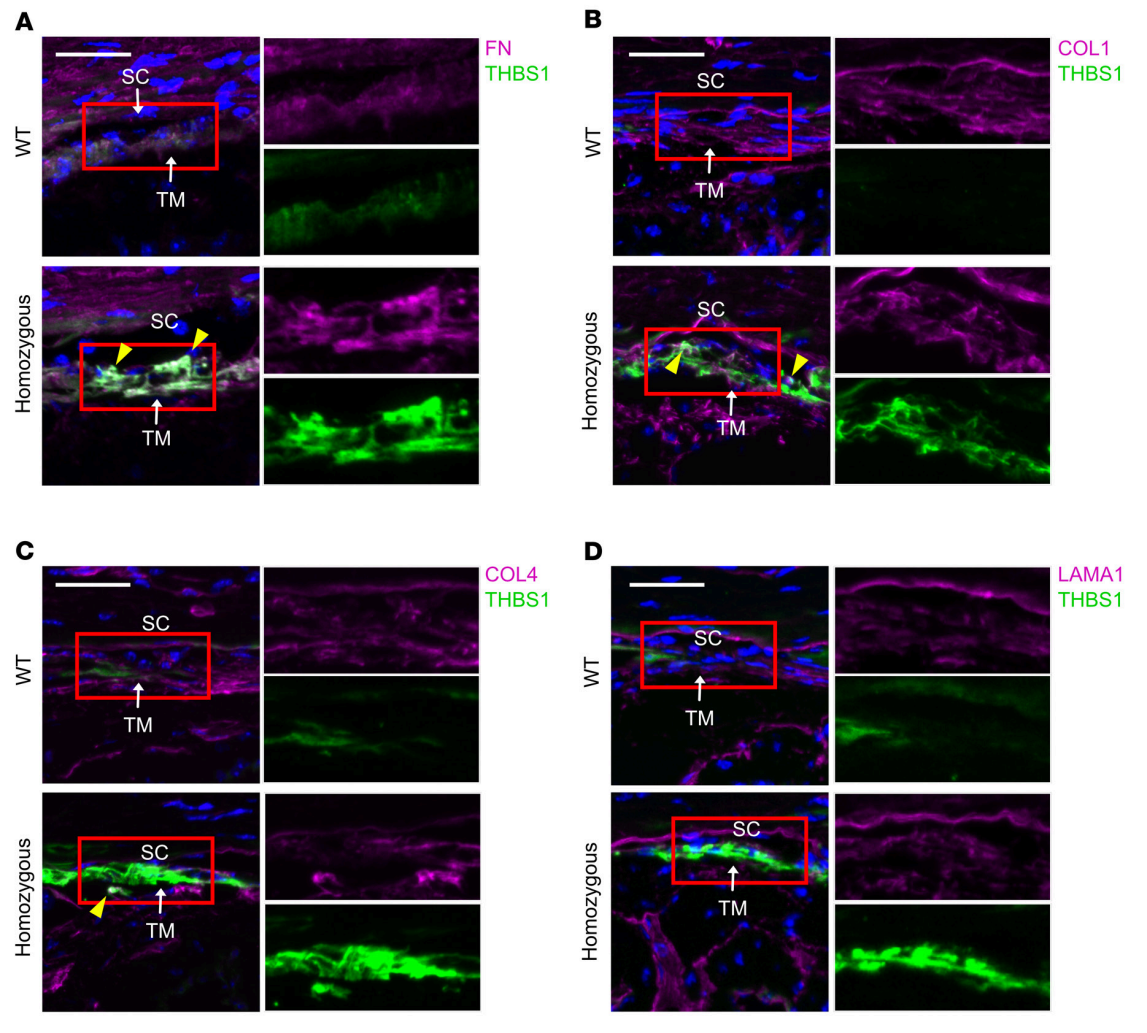
THBS1^R1034C^ recruits RN and COL1 to form ECM deposition in TM. Costaining for THBS1 (green) and (**A**) RN, (**B**) COL1, (**C**) COL4, or (**D**) LAMA1 (magenta) in cryosections of the iridocorneal angle region. THBS1^R1034C^ colocalized (yellow arrowheads) with FN and to a lesser extent with COL1, COL4, and LAMA1. Nuclei were stained with DAPI. Scale bars: 20 μm. Insets: ×1.6.

**Figure 9:**
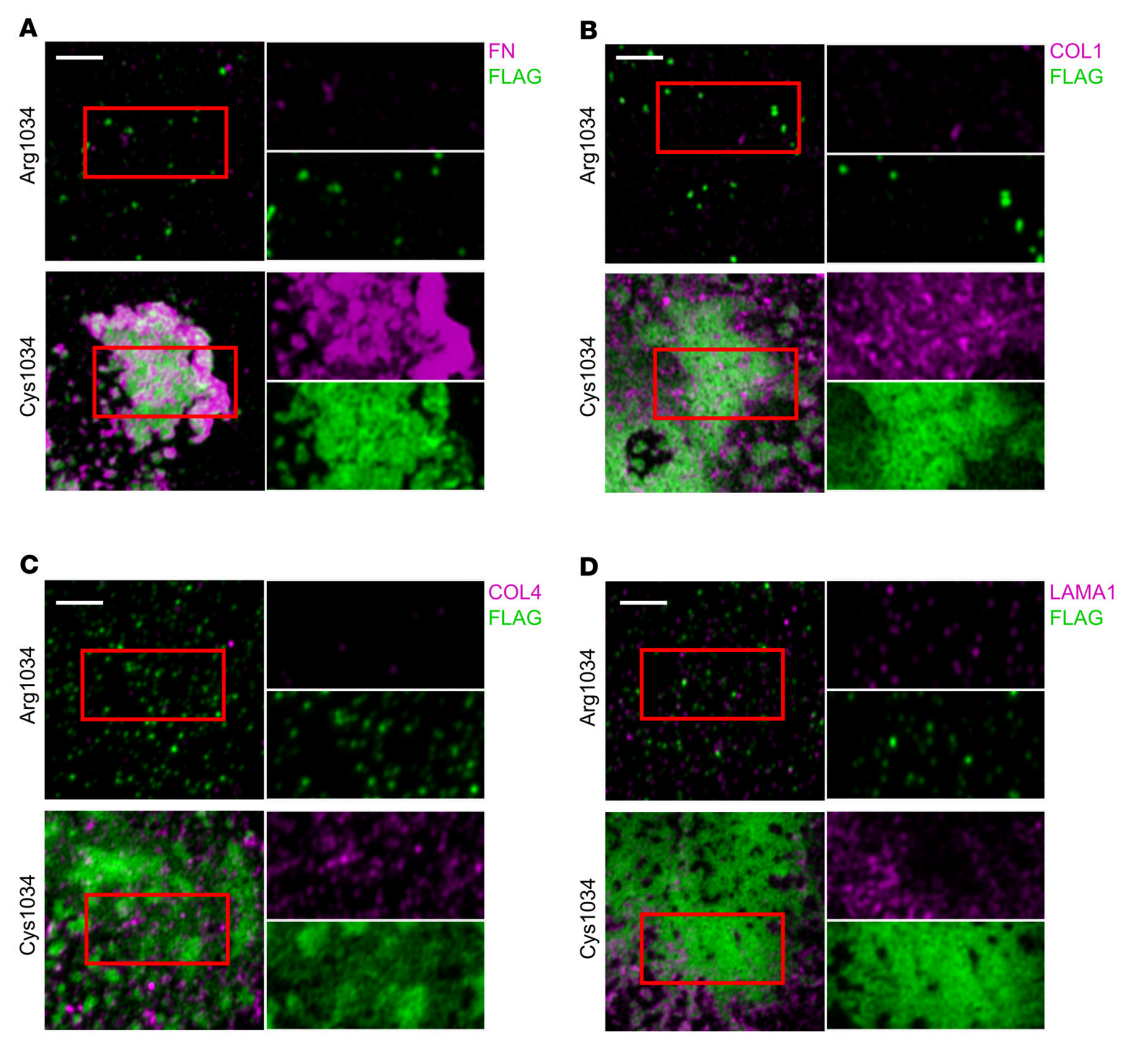
THBS1^R1034C^ recruits RN and COL1 to form ECM deposition in COS-7 cells. Costaining for THBS1 (green) and (**A**) FN, (**B**) COL1, (**C**) COL4, and (**D**) LAMA1 (magenta) in the ECM in COS-7 cells. Consistent with the findings in TM, there was strong accumulation of FN, which colocalized with THBS1^C1034^. Additionally, there was moderate accumulation of COL1. Scale bars: 2 μm. Insets: ×1.6.

In the JCT, THBS1 accumulation could also cause retention of other ECM proteins. Using immunostaining, we showed that fibronectin (FN) and collagen type I (COL1) accumulated and colocalized with THBS1 in the JCT (Figure 8, A and B) and in extracellular deposits in COS-7 cells (Figure 9, A and B) and in primary TM cells isolated from mutant mice (Supplemental Figure 12). In contrast, we did not observe increased expression or colocalization of collagen type IV (COL4) or laminin subunit α 1 (LAMA1) with THBS1 in mutant mice (Figure 8, C and D) or in extracellular deposits (Figure 9, C and D). Those findings indicated that mutant THBS1 selectively accumulated interstitial ECM components, predominantly FN, to form extracellular aggregates. R1034 is located in a protein region that binds CD47 (Figure 1), however, neither overexpression nor inhibition of CD47 expression in COS-7 cells affected the accumulation of THBS1 after transfection with THBS1-expressing plasmids, suggesting that CD47 was not involved in the formation of mutant protein deposits (Supplemental Figure 13). Compared with WT, we observed an increase in active TGF-β in purified TM cells from mutant mice, suggesting that accumulated THBS1 could lead to increased TGF-β activation (Supplemental Figure 14).

## Discussion

Here, we show that *Thbs1^R1034C^* mice developed elevated IOP, reduced outflow facility, and loss of RGCs consistent with a glaucoma phenotype. Mutant mice also exhibited extensive extracellular accumulation of THBS1 in the TM, with abnormal JCT morphology, a structure critically involved in ocular fluid outflow and IOP regulation. Using a cell-based assay, we show that extracellular mutant protein deposition was correlated with theoretical protein instability induced by variations in amino acid sequences. The extracellular aggregates included other ECM proteins, suggesting a disruption of normal ECM homeostasis due to an accumulation of mutant THBS1.

Although the *Thbs1^R1034C^* mice demonstrated sustained IOP elevation, they did not have the very high IOP observed in human carriers of the mutation. One explanation for this difference is that mice have a greater dependence on the “unconventional” uveoscleral outflow pathway, and our data suggest that this uveoscleral outflow pathway was not impaired by THBS1 deposition (Supplemental Figure 7). Previous studies suggest that up to 75% of aqueous outflow in mice occurs through the uveoscleral outflow pathway, while only 10%–15% of aqueous humor is removed by this pathway in humans (1). Therefore, we hypothesize that the observed difference in IOP between the mutant mice and human mutation carriers is due to the greater use of the uveoscleral outflow pathway in the mutant mice, however, further study will be necessary to confirm this hypothesis.

The observed extracellular protein aggregation in mutant THBS1 mice contrasts with the intracellular protein aggregation associated with childhood glaucoma–causing mutations in 2 other TM extracellular proteins: MYOC (24) and EFEMP1 (13). Human glaucoma–causing mutations have not yet been reported for any of the other TM ECM proteins, and this is the first observation, to our knowledge, of extracellular protein aggregation associated with glaucoma. Genetic disruption of *SVEP1*, a large ECM protein expressed in the TM, has been shown to cause a severe congenital glaucoma phenotype in mice (29). A *SVEP1* missense allele has also been shown in humans to modify the severity of TEK disease–causing mutations (30), and a common *SVEP1* missense allele is also associated with adult-onset primary open-angle glaucoma (POAG) (5). Protein-coding variants affecting various members of the *ADAMTS* family (a disintegrin and metalloproteinase with THBS1 motifs) have been associated with a range of glaucoma-related phenotypes in dogs (31, 32), however, in humans *ADAMTS* mutations cause syndromic developmental disorders not directly related to glaucoma, including Weill Marchesani syndrome, a systematic disease causing small lenses, short stature, and joint abnormalities (33). To our knowledge, the patients with *THBS1* missense alleles described here developed glaucoma without any clinical evidence of systemic disease. In our mouse model, we also note that the mutant THBS1 appeared normal in other organs (Supplemental Figure 15). It is possible that the dynamic turnover of the TM ECM was uniquely affected by the THBS1 mutations. Future work will be necessary to address the specific role of THBS1 in the TM ECM.

*THBS1* R1034, the residue modified in all 3 families, is a highly evolutionarily conserved amino acid, indicating a critical role in protein function. The observation that *Thbs1*-null mice have lower IOP (20) suggests that alteration of R1034 leads to a gain-of-function or dominant-negative mechanism. Our results showed colocalization of the THBS1 deposits with FN both in vivo and in vitro, suggesting a possible contribution of FN aggregation to decreased aqueous outflow. THBS1 is known to activate TGF-β in TM ECM, which can induce the formation of detrimental FN fibrils (34). As the TGF-β activation domain is in the N-terminal, it is possible that aggregated mutant THBS1 is capable of TGF-β activation, and we provide data on increased TGF-β activation in primary TM cells from mutant mice that support this hypothesis. Additionally, ECM turnover is an important feature of healthy TM ECM (18, 35), and the accumulation of THBS1 deposits may prevent normal homoeostasis. R1034 is also the first amino acid of a highly conserved 8–amino acid sequence that is involved in THBS1 interaction with CD47 (Figure 1). CD47 is a ubiquitously expressed transmembrane protein that plays multiple roles in fundamental cellular functions including phagocytosis, proliferation, and adhesion (36). In the eye, CD47 influences immune responses and inflammation (37), and in the TM, it has been reported to influence the development of the cross-linked actin network (CLAN), which, in glaucomatous TM cells, has been shown to contribute to reducing outflow facility through the TM (38). However, in our study, we did not identify a definitive role for CD47 in the accumulation of mutant THBS1. Other proteins and molecules may also be involved in the underlying disease mechanism, and these may be discovered through future work.

*THBS1* mutations have not been identified as a cause of any other human disease, despite apparent selective pressure against gene inactivation (39). A common *THBS1* variant (N700S, minor allele frequencies of 0.12 in White Europeans) may modify the risk of myocardial infarction (40) and may contribute to small infant size (41). A recent report suggests that this variant may be more frequent in TM cell lines from patients with glaucoma compared with controls (42), however, in our cell-based assay, this variant did not cause extracellular protein deposition (Supplemental Figure 16). Moreover, an association of N700S, or any other variant in the *THBS1* genomic region, has not been identified for any form of glaucoma in sufficiently powered GWAS (5–7). Interestingly, a recent analysis of WES data from the UK Biobank (43) identified a nominally significant gene-based association (*P* = 0.01, sequence kernel association test [SKAT]) for *THBS1* rare coding variants and POAG (ICD-10 diagnosis code H40), suggesting that further study of rare *THBS1*-coding variants in adult-onset POAG might be of interest.

In this study, we report on 3 unrelated and ethnically diverse families with congenital glaucoma, in which the affected members are carriers of missense alleles involving the same *THBS1* R1034 residue. The recurrence of R1034C in families 1 and 2 as well as the identification of R1034S in the affected children in family 3 strongly support a role for these missense alleles in disease pathogenesis, especially given the diverse ethnic backgrounds of the 3 families and the absence of these variants in any large sequence database. However, although R1034C segregates completely with disease in family 1, the fathers in families 2 and 3 have not been diagnosed with early-onset disease, suggesting that these *THBS1* missense alleles may result in disease with variable expressivity and severity, similar to other childhood glaucoma resulting from the genes *MYOC*, *EFEMP1*, *TEK*, *ANGPT1*, and *MAB21L1* (a gene causing microphthalmia) (9, 10, 12, 13, 21). Variable disease severity in *MYOC* mutation carriers has recently been shown to be affected by polygenic effects (44), and glaucoma variation caused by some TEK mutations may be due to the modifying effects of an SVEP1 variant (30). In this study, our data did not suggest that other modifying factors could contribute to disease in these families, however, further study using whole-genome sequencing (WGS) or other approaches could be of interest.

Our results suggest that *THBS1* mutations contributed to the development of some cases of early-onset glaucoma, expanding the number of genes responsible for this devastating form of glaucoma. The discovery of genes causing childhood glaucoma has important clinical implications, including the use of genetic testing to identify carriers of disease-causing mutations and to inform genetic counseling for affected families, allowing for the appropriate surveillance and treatment plans for mutation carriers (45). Treatment initiated at the early stages of disease can delay irreversible optic nerve degeneration and provide the best chance that an affected child will maintain useful sight throughout their lifetime.

In summary, we have identified *THBS1* missense alleles in children severely affected by early-onset glaucoma in 3 unrelated and ethnically diverse families. Our results suggest that the disease-associated variants cause significant ECM protein aggregation due to mutant THBS1 misfolding. These results further support a role for THBS1 in the regulation of intraocular fluid dynamics and IOP and provide new opportunities for genetic testing and therapeutic intervention.

## Methods

Further details on the methods are provided in Supplemental Methods.

### Exome sequencing.

For the family from the United States, WES was performed with the Agilent SureSelect V4 + UTR (74.4 Mb target sequence) library preparation kit and the Illumina HiSeq 2000 sequencer. Reads were aligned to the human reference sequence (Ensembl Genome browser hg19) with BWA (46) (version 0.6.2-r126), and SAMtools (47) (version 0.1.18 or r982:295) was used to remove potential duplicates and make initial SNP and indel calls. Resulting variant calls were annotated using our custom human resource (48). Overall, between 22,254 and 19,200 exonic sequence alterations were identified in each family member. The average exon sequencing depth was 110×, and 99% of the targeted region was covered with a minimum read depth of 40×. Confirmation of WES variants and *THBS1* screening of the US cohort was completed using Sanger sequencing. For the Australian families, genomic DNA was extracted from peripheral whole blood using the QiaAmp DNA Blood Maxi Kit (QIAGEN) following the manufacturer’s protocols. Samples were subjected to exome capture with the Agilent SureSelect V4 kit and sequenced on an Illumina HiSeq 2000, with data aligned and processed as previously described (11).

### Generation of Thbs1^R1034C^-transgenic mice.

We designed a CRISPR-mediated mouse germline knockin strategy (49) to create a point mutation (chr2:118123372 C>T, R1034C) in *Thbs1* using a single guide RNA (gRNA) (TCCACATCACAACGTAGAAG). Microinjection into C57BL/6J embryos (JAX stock no. 000664, The Jackson Laboratory) was done by the Boston Children’s Hospital (BCH) IDDRC Mouse Gene Manipulation Core. Briefly, synthetic crRNA and tracrRNA (Integrated DNA Technologies [IDT]) was mixed at 25 ng/μL to form a duplex at 95°C for 5 minutes and then cooled at room temperature for 20 minutes. The Cas9 protein (IDT) at 100 ng/μL was incubated with the crRNA:tracrRNA duplex at 37°C for 15 minutes. The cocktail of crRNA:tracrRNA:Cas9 protein was introduced by pronuclear microinjection into 0.5 dpc embryos and transferred into recipient females. For the purpose of genotyping, genomic DNA was extracted from a tail biopsy, and the region flanking the mutated target was amplified using PCR (primers are listed in Supplemental Table 1). Mutants were identified by the digestion of PCR product using PvuII (New England BioLabs) and confirmed by Sanger sequencing. We backcrossed our mutant mice to the WT C57BL/6J strain twice to exclude the possibility of any off-target effects prior to phenotyping. To further assess off-target effects, the gRNA sequence (TCCACATCACAACGTAGAAG) was blasted across the WT C57BL/6 genome. Two genes were considered possible targets: *Thbs2* (2 bp mismatch) and *Hk1* (2 bp mismatch). These regions containing the gRNA recognition sites were amplified using PCR with flanking primers (Supplemental Table 1) followed by Sanger sequencing, which showed normal sequences for both regions in the mutant mice.

### Mouse husbandry.

Age-matched C57BL/6J mice (JAX stock no. 000664) were purchased from The Jackson Laboratory. Housing rooms were kept at 21°C with a 12-hour light (6 am to 6 pm)/12-hour dark cycle. All mice were given free access to a standard diet (LabDiet 5K52 formulation with 6% fat) and water.

### IOP measurement.

IOP was measured using a Tonolab rebound tonometer (iCare Finland). After anesthesia by isoflurane for 5 minutes, the tonometer probe was placed perpendicularly to the center of the cornea to obtain 6 consecutive measurements, which were then averaged. Three averaged measurements were obtained for each eye. All IOP measurements were performed between 10 am and 11 am.

### Slit lamp biomicroscopy.

Mice under isoflurane anesthesia were photographed under a slit lamp microscope (Nikon FS-2) mounted with a single-lens reflex camera. For en face imaging of the cornea, the light beam was set at the maximum width (14 mm) with the illumination angle at 30°. For assessment of the anterior chamber depth, the light beam was adjusted to 0.5 mm, and the illumination angle was set at 45°. A photo was taken when the light vertically passed through the pupil.

### Outflow facility measurements.

The outflow facility was measured using a constant pressure perfusion system as previously described (50). Briefly, the anterior chamber was intubated by a 33 gauge needle that was then attached to a water column to provide perfusion pressure, which was determined by the fluid height in the column. A pressure transducer (catalog BLPR2, World Precision Instruments), along with a data acquisition device (catalog LabTrax-4, World Precision Instruments) and a transducer amplifier (catalog SYS-TBM4M, World Precision Instruments), was laterally attached to the system as a real-time monitor to ensure constant perfusion pressures. Sterile Dulbecco’s PBS (0.1 M) with the addition of 5.5 mM d-glucose (DBG) was used as a perfusion solution. Under anesthesia (avertin, 250 mg/kg), the anterior chamber was intubated by a 33 gauge needle through the mid-peripheral cornea. The outflow facility (*c*, μL/min/mmHg) was determined by the linear relationship between the flow rate (*r*, μL/min) and IOP (*p*, mmHg), based on the Goldmann equation, where Pe is the episcleral venous pressure. 

 (Equation 1) 
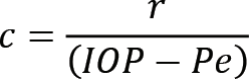


By adjusting the height of fluid in the column, the perfusion pressure was varied (8 mmHg, 16 mmHg, 24 mmHg, and 32 mmHg). Under each pressure, the perfusion volume (*v*) was determined by the change in fluid height. 

 (Equation 2) 
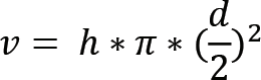


The averaged flow rate (*r*, μL/min) under each pressure was obtained by measuring the perfusion volume (*v*) over time (*t*). 

 (Equation 3) 
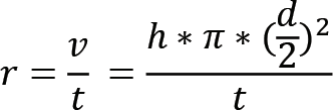


Finally, the outflow facility (*c*, μL/min/mmHg) was calculated by linear regression analysis between the pressure (*p*, mmHg) and the corresponding flow rate (*r*, μL/min).

### RGC counts.

RGC counts were determined after immunofluorescence staining for BRN3A in whole-mounted retina. Central (1 mm to the optic nerve head) and peripheral (2 mm) retinas were assessed separately. To achieve reliable counting, 6 areas of interest (AOI) (0.2 mm × 0.2 mm) in the central retina and 12 AOIs in the peripheral retina for each eye were sampled and averaged, respectively. RGC densities are presented as cell counts per mm^2^.

### Corneal micropocket assay.

The corneal micropocket assay was performed as previously described (51). Briefly, once the mouse was anesthetized (avertin, 250 mg/kg), a slow-release pellet containing 20 ng basic FGF (catalog 100-18B, Peprotech) was implanted into the stromal layer of the cornea. Five days later, the corneas were harvested and stained with anti-CD31 and anti-LYVE1 antibodies to visualize limbal blood vessels and lymphatic vessels, respectively. Quantification was performed using AngioTool according to a previously published protocol (52, 53).

### Quantification of the SC area.

The measurement of SC areas was based on the whole-mounted staining for CD31 using the threshold function in ImageJ (NIH). Measurements were performed at 4 quadrants of the SC per cornea under a ×20 lens field (0.501 mm^2^).

### TEM analysis.

To examine the ultrastructure of the TM and surrounding ECM, eyes from age-matched WT and mutant mice were enucleated for TEM analysis. For ultrastructural studies, eyes were perfusion fixed with 2.5% glutaraldehyde and 2.5% paraformaldehyde in 0.1 M sodium cacodylate buffer (pH 7.4) before enucleation and kept in the same fixation. For the pre-embedding gold particle immunolabeling TEM studies, the iris, lens, and posterior segment were carefully removed without damaging the ciliary body or iridocorneal tissue. The anterior segments were then immersion fixed in 4% paraformaldehyde and 0.1% glutaraldehyde in 0.1M sodium phosphate buffer (PB), pH 7.4, at room temperature for 1 hour. To quench free aldehyde groups, the eyes were washed 3 times with 20 mM glycine in 0.1 M PB for 10 minutes. The samples were then blocked and permeabilized with 10% normal goat serum, 1% BSA, and 0.5% Triton X-100 in 0.1 M PB at room temperature for 3 hours. Anti-THBS1 antibody (catalog ab85762, Abcam) was used as the primary antibody (5 μg/mL). After 3 days of incubation at 4°C, the samples were washed 3 time with 0.1 M PB and incubated with 5 nm gold particle–conjugated Protein A (catalog GA1053, BOSTER) at room temperature for 1 hour. Afterwards, the tissues were washed extensively with PBS (1×) buffer and postfixed with 2.5% glutaraldehyde and 0.1 M sodium cacodylate buffer (pH 7.4). Both the labeled and unlabeled samples were then washed in 0.1 M cacodylate buffer and postfixed with 1% osmium tetroxide (OsO_4_)/1.5% potassium ferrocyanide (KFeCN6) for epon embedding. Afterwards, they were washed in water 3 times and incubated in 1% aqueous uranyl acetate for 1 hour followed by 2 washes in water and subsequent dehydration in alcohol (10 min each; 50%, 70%, 90%, and 2 × 10 min at 100%). The samples were put in propylene oxide for 1 hour and infiltrated overnight in a 1:1 mixture of propylene oxide and TAAB Epon (Marivac Canada). The following day, the tissue was embedded in TAAB Epon and polymerized at 60°C for 48 hours. Ultrathin sections (80 nm) were cut on a Reichert Ultracut-S microtome and collected on copper grids. After staining with lead citrate, samples were examined on a JEOL 1200 EX TEM.

### Immunofluorescence microscopy.

Immunofluorescence (IF) staining of the iridocorneal angle was performed using cryosections and whole-mounted eyes. Enucleated eyes were fixed with 4% paraformaldehyde (catalog 15710, Electron Microscopy Sciences) in 0.1 M PB at room temperature for 1 hour. For cryosection staining, eyeballs were dissected to remove the central portion of the cornea and lens. Care was taken to not damage the iridocorneal tissue or retina. The eyes were then washed in 0.1 M PB thoroughly and dehydrated in 30% sucrose solution/0.1 M PB in 4°C for 3 hours. Other tissues (colon, kidney, liver, and lung) were fixed in 4% paraformaldehyde overnight before the sucrose dehydration. After embedding in frozen sectioning medium (Tissue-Tek O.C.T Compound, Sakura Finetech), vertical cryosections were made at 12 μm and stored at –20°C until use. The sections were rehydrated with 0.1 M PB and then blocked and permeabilized for 1 hour with 10% normal goat serum (catalog 50062Z, Thermo Fisher Scientific), 1% BSA (catalog A5611-10G, MilliporeSigma), 0.5%Triton X-100, and 0.1 M PB. For biotinylated primary antibodies, samples were further blocked using an Endogenous Biotin-Blocking Kit (catalog E21390, Invitrogen, Thermo Fisher Scientific) according to the manufacturer’s instructions. Slices were incubated with a primary antibody overnight at 4°C in blocking solution. Subsequently, they were washed 3 times in 0.1 M PB and incubated with a secondary antibody at room temperature for 1 hour. After thorough washing in 0.1 M PB, the slides were mounted in DAPI Fluoromount-G medium (catalog 0100-20, SouthernBiotech). For whole-mount staining, the tissues were dissected from eyeballs and washed briefly in 0.1 M PB. The tissues were then blocked and permeabilized in 10% normal goat serum, 1% BSA, 0.5%Triton X-100, and 0.1 M PB at room temperature for 3 hours. Samples were incubated with a primary antibody for 3 days at 4°C. Subsequently, they were washed 3 times in 0.5%Triton X-100 and 0.1 M PB and incubated with a secondary antibody at room temperature for 3 hours. After thorough washing in 0.5% Triton X-100 and 0.1 M PB, the corneas and retinas were cut radially and flat-mounted with Fluoromount-G medium (catalog 0100-01, SouthernBiotech). Images were taken with a Zeiss LSM880 confocal scanning microscope. The following primary antibodies were used: anti-THBS1 antibody (5 μg/mL, catalog ab85762, Abcam); anti–THBS1 biotinylated antibody (5 μg/mL, A6.1, catalog MA5-13395, Thermo Fisher Scientific); anti–FN biotinylated antibody (10 μg/mL, catalog ab6584, Abcam); anti-COL1 antibody (10 μg/mL, catalog ab34710, Abcam); anti–collagen type IV antibody (1 μg/mL, catalog 55131-1-AP, Proteintech); anti-laminin antibody (10 μg/mL, catalog NB300-144, Novus Biologicals); PE-conjugated anti-CD31 antibody (4 μg/mL, catalog 553373, BD Pharmingen); anti–LYVE-1 biotinylated antibody (4 μg/mL, catalog BAF2125, R&D Systems), and anti-BRN3A antibody (4 μg/mL, catalog ab245230, Abcam). The following secondary antibodies were used: goat anti–rabbit IgG H&L highly cross-adsorbed secondary antibody (1:500 for section staining, 1:200 for whole-mount staining, Alexa Fluor Plus 647, catalog A32733, Thermo Fisher Scientific); goat anti–rabbit IgG H&L (1:500 for section staining, 1:200 for whole-mount staining, Alexa Fluor 488, catalog ab150077, Abcam); and streptavidin (1:500, Alexa Fluor 647, catalog S32357, Thermo Fisher Scientific).

### Western blot analysis.

Eyes were enucleated from adult THBS1-transgenic mice and age-matched C57BL/6J mice within 30 minutes of euthanasia. The cornea and iridocorneal tissue, including the TM, SC, iris, and ciliary body, were dissected under a stereo microscope. Protein was extracted by homogenization in T-PER Tissue Protein Extraction Reagent (catalog 78510, Thermo Fisher Scientific) along with the Halt Protease Inhibitor Cocktail (1:100 dilution, catalog 78429, Thermo Fisher Scientific). The protein concentration was determined with a Pierce BCA Protein Assay Kit (catalog 23225, Thermo Fisher Scientific). Total protein (30 μg) was loaded into a Bolt 4%–12%, Bis-Tris Protein Gel (NW04120BOX, Invitrogen, Thermo Fisher Scientific) and transferred onto a PVDF membrane. After transferring, membranes were briefly washed in TBS 1× buffer and 0.1% Tween-20, and then blocked with 5% nonfat milk, TBS 1× buffer, and 0.1% Tween-20 at room temperature for 1 hour. Next, they were incubated with the primary antibody at 4°C overnight. After 3 washes in TBS 1× buffer and 0.1% Tween-20, the membranes were then incubated with the secondary antibody at room temperature for 1 hour. The targeted proteins were visualized by SuperSignal West Pico PLUS Chemiluminescent Substrate (catalog 34579, Thermo Fisher Scientific), and imaged with a Molecular Imaging System (Bio-Rad). The following antibodies were used: anti-THBS1 antibody (1 μg/mL, catalog ab85762, Abcam); anti-BiP antibody (1:1,000 dilution, catalog 3177S, Cell Signaling Technology); anti-Grp94 antibody (1:1,000 dilution, catalog 20292S, Cell Signaling Technology); anti-IRE1α antibody (1:1,000 dilution, catalog 3294S, Cell Signaling Technology); anti-ERp72 antibody (1:1,000 dilution, catalog 5033S, Cell Signaling Technology); anti–β-actin peroxidase-conjugated antibody (1:7,000 dilution, catalog A3854-200UL, MilliporeSigma); and anti–rabbit IgG, HRP-linked antibody (1:10,000 dilution, catalog 7074S, Cell Signaling Technology).

### RNA isolation and quantitative real-time PCR.

The limbal area including the ciliary body was dissected for RNA isolation. Total RNA was extracted and purified using an RNeasy Plus Mini Kit (catalog 74134, QIAGEN). RNA quality was assessed with a NanoDrop spectrophotometer 8000 (Thermo Fisher Scientific). RNA (500 ng) was used to synthesize cDNA with a High-Capacity cDNA Reverse Transcription Kit (catalog 4368814, Applied Biosystems). A total of 50 ng cDNA and 500 nM primers were used for quantitative real-time PCR (qPCR) analysis with PowerUp SYBR Green Master Mix (catalog A25741, Applied Biosystems). The reaction was set up under the standard thermocycling mode in StepOnePlus (Applied Biosystems) using a uracil-DNA glycosylase (UDG) activation step of 50°C for 2 minutes, followed by an initial step of 95°C for 2 minutes and then 40 cycles (15 s at 95°C, 15 s at 55°C, 1 min at 72°C). The relative mRNA amounts were calculated using the ΔΔCt method normalized to the housekeeping gene *Gapdh*. The primers for qPCR analysis are listed in Supplemental Table 1.

### Prediction of protein stability.

The crystal structure of the human THBS1 C-terminal fragment (Protein Data Bank [PDB] entry 1UX6) (26) was used as the input structure for the empirical force field algorithm FoldX 5.0 (27). Before mutagenesis, the crystal structure was optimized using the “Repair object” command to minimize the free energy of the structure (ΔG, kcal/mol). Every mutant was constructed through the “Mutate residue” command. The stability change was determined by calculating the free unfolding energy difference (ΔΔG) between WT Thbs1 and the corresponding mutant. 

 (Equation 4)
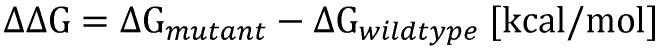


An averaged ΔΔG from 5 trails was used in this study. Figure 6 was made by PyMOL 2.3 using the minimized structures of THBS1^R1034^ and THBS1^C1034^.

### Isolation and culturing of mouse primary TM cells.

Mouse primary TM cells were isolated from *Thbs1^R1034C^* and age-matched C57BL/6J mice according to previously published protocol (53). Briefly, 2 μL sterile magnetic polystyrene microbeads (1.0% in wt/vol, catalog PMS-20-10, Spherotech) were injected intracamerally using a 33 gauge and needle and a Hamilton syringe. One week after the injection, eyes were harvested and disinfected in povidone-iodine (10%) for 2 minutes and washed in 0.1 M PB. Under a surgical microscope, the iris, lens, and posterior segment were removed, leaving the iridocorneal tissue intact. Tissues from at least 12 eyes were pooled and digested in 1 mL digestion medium (4 mg/mL collagenase A and 4 mg/mL BSA in 0.1 M PB) for 2 hours at 37°C. To remove tissue fragments, the digestion medium was filtered through a 100 μm cell strainer into a 1.5 mL tube. The tube was attached to a magnet to pellet the TM cells from the remaining medium. DMEM (1 mL, high-glucose supplemented with 10% FBS and antibiotics) was added to resuspend the cell pellet. This washing step was repeated twice. Finally, the TM cells were resuspended in 100–200 μL DMEM and seeded onto a 96-well plate. TM cells were maintained in DMEM and subculture at a ratio of 1:4.

### ECM deposition in vitro.

The following plasmids containing cDNA coding for THBS1 with the N-terminal FLAG tag were purchased from Sino Biological for mutagenesis: human *THBS1* cDNA ORF clone FLAG tag (catalog HG10508-NF, Sino Biological) and mouse *Thbs1* cDNA ORF Clone FLAG tag (catalog MG50655-NF, Sino Biological). R1034 alteration was done using the Q5 Site-Directed Mutagenesis kit (catalog E0554S, New England BioLabs). The primers for mutagenesis are listed in Supplemental Table 1. Sanger sequencing was used to confirm accurate mutagenesis. WT *Thbs1* and *Thbs1* mutants were then expressed in COS-7 cells (catalog CRL-1651, ATCC) and maintained in high-glucose DMEM supplemented with 10% FBS and penicillin-streptomycin. Cells (2 × 10^5^) were plated in 6-well plates the day before transfection. Plasmid DNA (1.6 μg) was transfected using PolyFect Transfection Reagent (catalog 301105, QIAGEN) following the manufacturer’s instructions. After overnight incubation, cells were detached and re-seeded into 4-chamber slides and incubated for another day to allow for ECM deposition. To visualize cell-derived ECM, cells were removed by 20 mM ammonium hydroxide as previously described (54). Briefly, cells were immersed in 20 mM ammonium hydroxide for 5 minutes with gentle shaking. Subsequently, chamber slides were washed 3 times with distilled water followed by protein immunofluorescence staining (described above). An absence of DAPI and phalloidin (F-actin) staining signals indicated successful removal of cellular components. The deposition of WT and mutant THBS1 was quantified using ImageJ to measure the fluorescence intensity of anti-FLAG staining. The primary antibodies used were as follows: anti-FLAG antibody (1:1,000 dilution, catalog 8146S, Cell Signaling Technology); anti-FN antibody (10 μg/mL, catalog ab6584, Abcam); anti-COL1 antibody (10 μL/mL, catalog ab34710, Abcam); anti-laminin antibody (10 μg/mL, catalog NB300-144, Novus Biologicals); and anti-COL4 antibody (1 μg/mL, catalog 55131-1-AP, Proteintech). The secondary antibodies used were as follows: goat anti–mouse IgG H&L (1:500, Alexa Fluor 488, catalog ab150113, Abcam) and goat anti–rabbit IgG H&L Highly Cross-Adsorbed Secondary Antibody (1:500, Alexa Fluor Plus 647, catalog A32733, Thermo Fisher Scientific).

### Statistics.

The outflow facility for each eye was determined by the slope of the regression line through linear regression analysis between the flow rate and the corresponding perfusion pressure. The correlation between computational stability changes (ΔΔG) and THBS1 deposition in ECM were evaluated by linear regression analysis. Longitudinal IOP data for WT and mutant mice were analyzed using a mixed-effects model. RGC density at central and peripheral retinas between WT and homozygous were analyzed by 2-way ANOVA. The numbers of optic nerve axons, outflow facility, and mRNA level of *Thbs1* were assessed by 2-tailed *t* test. The rest of the data including weight of the mice, SC size, corneal blood vessel and lymphatic vessel area, and ECM deposition under TM and COS-7 cells were assessed by 1-way ANOVA. All the analyses were performed in GraphPad Prism, version 8.0.2 (GraphPad Software). A *P* value of less than 0.05 was considered statistically significant. Data are represented in the figures as the mean ± SEM.

### Study approval.

All animal experiments were approved by the IACUC of Boston’s Children’s Hospital. Participants and their families were recruited from multiple international centers, which had each received study approval from their respective IRBs: Massachusetts Eye and Ear Infirmary Human Studies Committee; Southern Adelaide Clinical Human Research Ethics Committee, Flinders Medical Centre, Bedford Park, South Australia, Australia; and Royal Victorian Eye and Ear Hospital, East Melbourne, Victoria, Australia. Written informed consent for study participation was obtained from the participants or their parents. All animal procedures performed in this study complied with the Association for Research in Vision and Ophthalmology (ARVO) Statement for the Use of Animals in Ophthalmic and Vision Research and were approved by the IACUC of Boston Children’s Hospital (approval no. 19-03-3884R).

## Author contributions

RJD and JLW conceived, designed, supervised the study and wrote the manuscript. HF designed and performed in vivo and in vitro experiments, analyzed data, and wrote the manuscript. ERC and AEB designed and performed in vitro experiments, analyzed data, and contributed to the manuscript. OMS, JLW, and JEC contributed human samples and clinical data, performed genetic analysis, and contributed to the manuscript. LSWK, SES, and JBR contributed human samples and clinical data. All authors reviewed and approved the manuscript.

## Supplementary Material

Supplemental data

## Figures and Tables

**Table 1:**
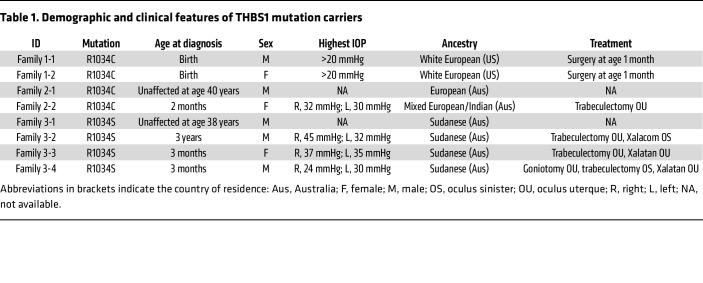
Demographic and clinical features of THBS1 mutation carriers

## Version 1 12/01/2022

Electronic publication
