# Supplementary material for: Thrombospondin 1 missense alleles induce extracellular matrix protein aggregation and TM dysfunction in congenital glaucoma
Source: J Clin Invest. 2022 Dec 1;132(23):e156967. doi: 10.1172/JCI156967 (PMC9711877; doi:10.1172/JCI156967)
Supplement: Supplemental data [file jci-132-156967-s029.pdf]

**Supplemental Material for:**

# Thrombospondin-1 missense alleles induce extracellular matrix protein aggregation and trabecular meshwork dysfunction in congenital glaucoma

Haojie Fu, Owen M. Siggs, Lachlan S.W. Knight, Sandra E. Staffieri, Jonathan B. Ruddle, Amy E. Birsner, E. Ryan Collantes, Jamie E. Craig, Janey L. Wiggs, Robert J. D'Amato

**This file contains:**

**1) Supplemental Methods**

**2) Supplemental Table 1**

**3) Supplemental Figures 1-16**

**4) References**

# Supplemental Methods

## Cumulative IOP

The cumulative IOP difference for each animal was calculated according to the following equation:

$$\text{Cumulative IOP difference (mmHg)} = \sum (IOP_{Mutant} - IOP_{Wildtype})_N$$

N is the number of IOP measurements taken per animal throughout the study (8 for *Thbs1*$^{R0134C}$ homozygous mice and 6 for heterozygous mice over 8 weeks).

## Measurement of uveoscleral outflow in mice.

Uveoscleral outflow was measured using a modification of a previously published method (1). Briefly, 2mg/ml of Fluorescein labeled dextran was prepared from 25mg/ml stock (D1822, Thermo Fisher Scientific). Six months old *Thbs1*$^{R0134C}$ homozygous and age-matched wildtype mice were used for the experiments. The anterior chamber was perfused at 16mmHg pressure for 1 hour. Then, the choroid and sclera tissue complex were dissected, homogenized, and centrifuged at 2700g for 5 minutes. The dextran fluorescent density was determined from the supernatant by a plate reader. The exact amount of dextran was calculated based on the dextran standard that was prepared from the serial dilutions of perfused solution.

## Quantification of optic nerve axons.

After enucleation, the optic nerve was cut approximately 1 mm behind the globe and fixed as described above. For degeneration axon counting, random micrographs (1000X magnification, 44.8 μm * 68.18 μm) were taken from different regions of the optic nerve. Degenerating axons were identified by one of the following structures: condensed electron opaque axoplasm, empty swollen axonal myelin sheath, myelin sheath filled with cellular debris, or destruction of the myelin sheath. Axons were counted from 4-12 micrographs for each eye and the numbers were averaged. For healthy axon counting, random micrographs (17.2 μm * 11.2 μm) were taken from different regions of the optic nerve. Axons were counted manually within the field. The number of axons counted from 4-12 micrographs for each eye were averaged and divided by 17.2 μm * 11.2 μm to obtain the mean axon numbers per μm$^2$.

## Detection of apoptosis in mouse trabecular meshwork.

In situ detection of trabecular meshwork cell death used cryosections from eyes from 6 months old *Thbs1*$^{R1034C}$ homozygous and age matched wildtype mice. TM cell death was detected based on the present of DNA breaks, which was labeled by TdT-

mediated dUTP-X nick end labeling (termed TUNEL) (12156792910, Millipore Sigma). For each mouse, 6 random sections of TM tissue were selected for the labeling. The total TUNEL$^{+}$ TM cells were counted and analyzed.

**CD47 overexpression and knockdown in COS-7 cells.**

The CD47 shRNA (tagged with GFP), and the CD47 overexpression vector (cDNA tagged with GFP) lentivirus particles along with the non-targeting and empty vector controls were purchased from Horizon Discovery. The COS-7 cells were seeded in 6-well plate at a confluency of 50%-60% prior to the transduction. The next day, cells were washed by PBS and lentivirus mix (DMEM basal media, lentivirus particles and 10ug/ml polybrene) was added directly to the cells. After 24 hours of transduction, the lentivirus mix was removed and fresh full DMEM media was added to the cells. Cells were then sorted by flow cytometry cell by GFP fluorescence to remove non-transduced cells. The expression of CD47 was determined by western blot using CD47 Monoclonal antibody, Cat No. 66304-1-Ig, Proteintech. Dilution, 1:1000. To examine effects on ECM deposition, COS-7 cells with either CD47 knockdown or CD47 overexpression, or wild type COS-7 cells (control) were transfected with either wildtype THBS1 or THBS1$^{R1034C}$ expressing plasmids followed by immunostaining as described in the Methods section.

**Detection of active TGF beta in primary trabecular meshwork cells.**

Primary TM cells from *Thbs1$^{R1034C}$* mutants and wildtype mice were seeded in 6-well plates. High glucose media without FBS was added when the cells reached a confluency of 70%-90%. The conditioned media was collected the next day. The cellular components were removed by 0.45 um filter. The media was then concentrated by a centrifugal filter unit (Millipore Sigma, Cat no. UFC901024) before being prepared for western blot using TGF beta1 Monoclonal Antibody (Dilution, 1:1000, clone F.888.7, Cat No. MA5-15065, Invitrogen).

**Sequencing of mRNA from wildtype and *Thbs1$^{R1034C}$* homozygous mice.** mRNA was isolated from mouse cornea containing the limbus area. The RNA isolation and cDNA synthesis were conduct the same way as qPCR experiments. A total of 50 ng cDNA along with 500nM primers was used to amplify a region flanking the mutation site (PrimeSTAR Max DNA Polymerase, Cat No. R045A, Takara). The primers are listed in the Supplemental Table 1. The PCR products were then gel purified for Sanger sequencing. The forward primer for PCR amplification was used as the sequencing primer.

## References

1. Millar J. C., et al. Strain and Age Effects on Aqueous Humor Dynamics in the Mouse. Invest Ophthalmol Vis Sci. 2015 Sep;56(10):5764-76.

## Supplemental Table 1. Primers

| | | Forward 5' to 3' | Reverse 5' to 3' |
|---|---|---|---|
| **Genotyping *Thbs1*$^{R1034C}$ mutation** | | | |
| | | ATCTGTCCATTGGACCTGTGGCCT | ACCTTGGTCGAAGTGCTGTCTCCT |
| **Identifying off-targets** | | | |
| | *Thbs2* | TGGGCCACCAGACCATCCATGT | TCTTCTCGCCCTGGACTGTGCA |
| | *Hk1* | CCATGACTCCATCTGGGATTT | CATGGAGGGAAACTCAGAAACTA |
| **qPCR** | | | |
| | *Thbs1* | TGGCCAGCGTTGCCA | TCTGCAGCACCCCCTCAA |
| | *Gapdh* | TCTCCCTCACAATTTCCATCC | GGGTGCAGCGAACTTTATTG |
| **Mutagenesis in mouse *Thbs1* cDNA** | | | |
| | R1034C | GTCCAGCAGCTGCTTCTACGT | TGGTAGCCGAATACAAAGC |
| | R1034G | GTCCAGCAGCGGCTTCTACGT | TGGTAGCCGAATACAAAGC |
| | R1034A | GTCCAGCAGCGCCTTCTACGTTG | TGGTAGCCGAATACAAAG |
| | R1034S | GTCCAGCAGCAGCTTCTACGT | TGGTAGCCGAATACAAAGC |
| | R1034Q | TCCAGCAGCCAGTTCTACGTTG | CTGGTAGCCGAATACAAAG |
| | R1034K | GTCCAGCAGCAAGTTCTACGTTGTG | TGGTAGCCGAATACAAAG |
| **Mutagenesis in human *THBS1* cDNA** | | | |
| | R1034C | GTCCAGCAGCTGCTTTTATGT | TGGTAGCCAAAGACAAATC |
| | N700S | GGCTGGCCCAGTGAGAACCTG | ATCCAGGTCTGTGTCCTCC |
| **Sequencing of mRNA** | | | |
| | | CCT GGA CTT GCT GTA GGT TAT G | GGG TTT CTC TAG CCC TTG TTT |

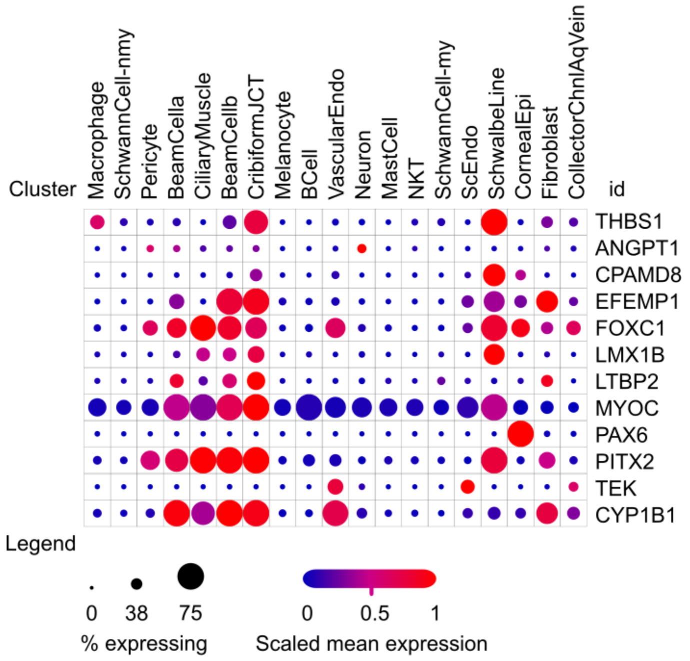


**Supplemental Figure 1. Dot blot plot showing single cell RNA sequencing data for *THBS1* and other known early onset glaucoma genes in cell types from human anterior ocular segment.** The plot was generated using data from the Broad Institute of MIT and Harvard's Single Cell Portal (https://singlecell.broadinstitute.org/single_cell) downloaded from "Cell atlas of aqueous humor outflow pathways in eyes of humans and four model species provides insight into glaucoma pathogenesis" by van Zyl et al, 2019. (Reference 22).

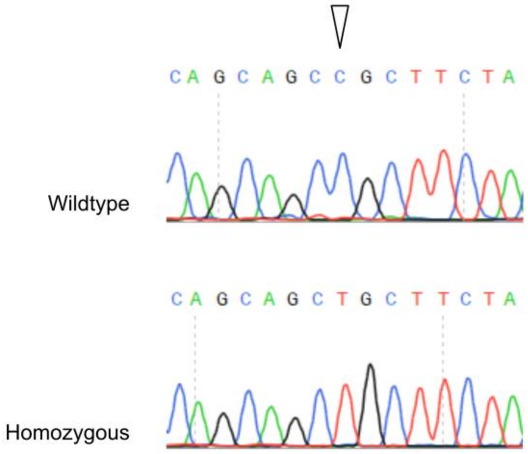


**Supplemental Figure 2. RNA sequence from *Thbs1*$^{R1034C}$ CRISPR mutant confirms the R1034C mutation.** mRNA was extracted from the cornea of *Thbs1*$^{R1034C}$ mutant and wildtype controls, followed by synthesis of single strand cDNA. Sanger sequencing of cDNA confirmed that a successful mutation (C→T, black arrowhead) was made in the mutant mice.

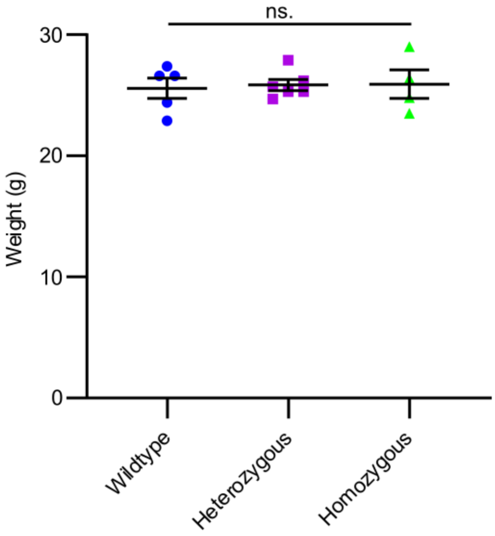


**Supplemental Figure 3. Weight for wildtype (N=4), and *Thbs1*$^{R1034C}$ heterozygous (N=5) and homozygous (N=6) mice.** Mice were weighed at 8 weeks of age. Data represent the means ± SEM. The heterozygous and homozygous *Thbs1*$^{R1034C}$ mice were littermates, the wildtypes were not. No significant difference in weight was observed (ANOVA, P=0.95). ns, not significant.

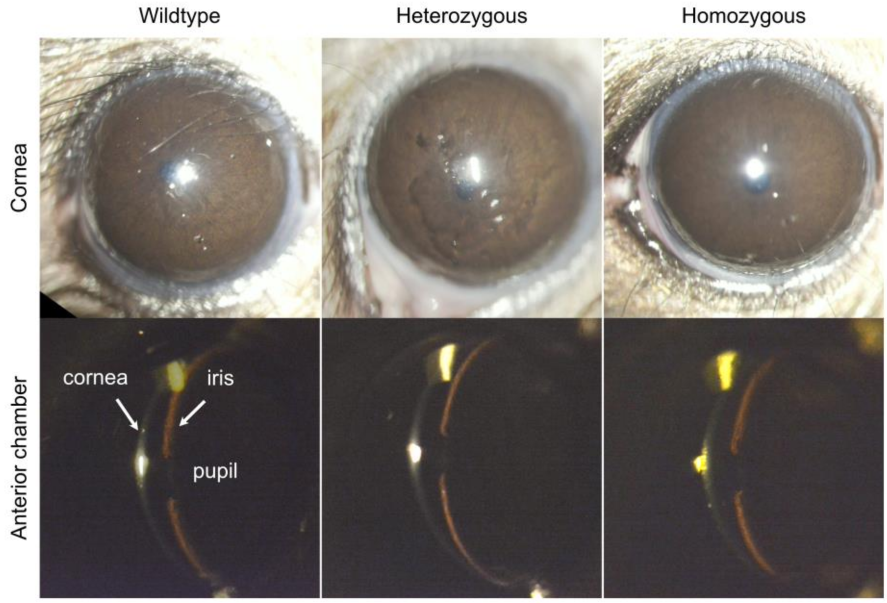


**Supplemental Figure 4. *Thbs1*$^{R1034C}$ mutants have normal anterior segment appearance.** Slit lamp-based biomicroscopy revealed normal appearing anterior segments (upper panel) and anterior chamber depth (lower panel) in 4-month-old heterozygous and homozygous *Thbs1*$^{R1034C}$ mutants.

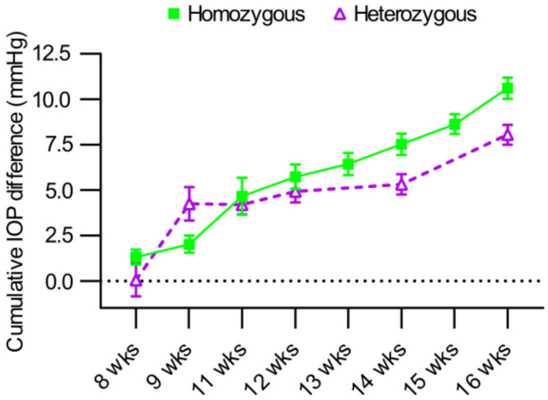


**Supplemental Figure 5. Cumulative intraocular pressure plot.** Data represent mean ± SEM. The black dotted line represents the wild type intraocular pressure.

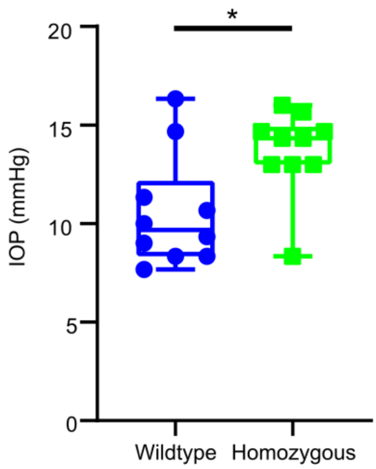


**Supplemental Figure 6. *Thbs1$^{R1034C}$* mutants exhibit elevated IOP compared to wildtype controls at age 7 months.** N=10, *p=0.012 as determined by unpaired t-test. The box boundaries represent the 25th to 75th percentiles and the line within each box is the mean value with the whiskers identifying the minimum and maximum values.

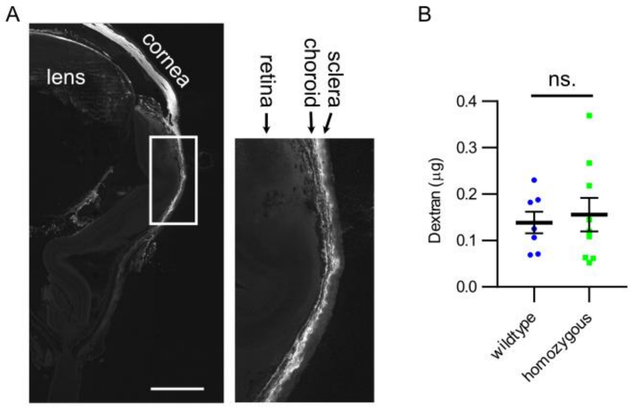


**Supplemental Figure 7. Uveoscleral outflow is not altered in *Thbs1*$^{R1034C}$ homozygous mutant mice compared to wildtype controls.** (**A**) 2mg/ml FITC-Dextran (70 kDa) was perfused through anterior chamber for 1 hour. Cryosection of the perfused eye (wildtype) showed the presence of fluorescent dextran in the choroid and sclera, representing the uveoscleral outflow pathway. The tissue complex was then dissected and homogenized. The uveoscleral outflow was calculated based on the fluorescence density of the supernatant. Scale bar, 500 µm. (**B**) There was no significant difference in uveoscleral outflow between *Thbs1* mutants and age-matched wildtype animals at 6 months of age. N=7, 9 for wildtype and homozygous, respectively, data represent the mean ± SEM. p=0.71 as determined by unpaired t-test. ns., not significant.

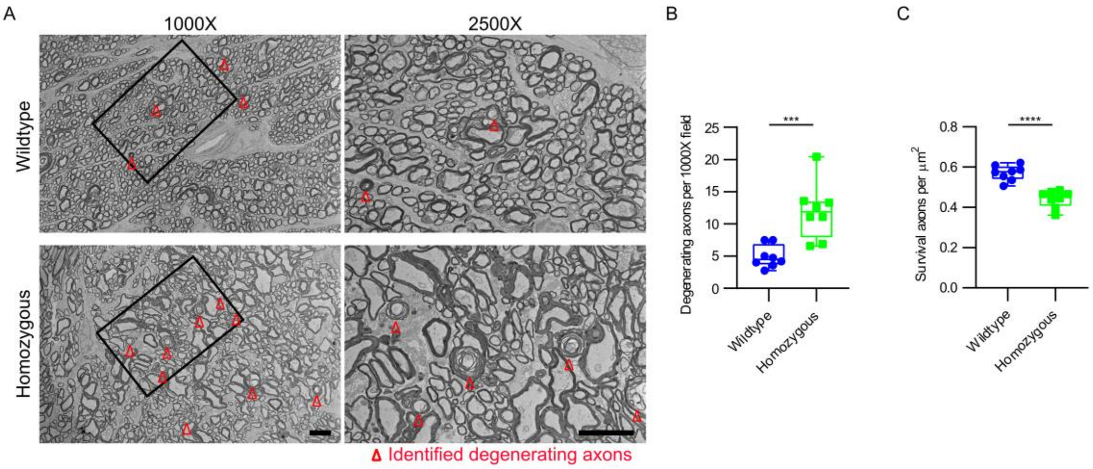


**Supplemental Figure 8. *Thbs1$^{R1034C}$* mutant mice have an increased number of degenerating ganglion cell axons and reduced number of surviving axons compared to wildtype controls.** (**A**) Electron microscopy of the optic nerve in cross section shows degenerating axons (red triangles) in 20-month-old wildtype and *Thbs1$^{R1034C}$* homozygous mice. Scale bar 5 μm. (**B**) Statistically significant increase in degenerating axons in mutant mice compared to wildtype animals (143.7% increase, N=8, (***p=0.0008, as determined by unpaired t-test). (**C**) Compared to the wildtype, survival axon bundle density was also less in the homozygous mice compared to the wildtype (22.8% reduction N=8, ****p<0.0001, as determined by unpaired t-test). Of note, these results are consistent with the findings from the retinal ganglion cell counting shown at Figure 3-E. The box boundaries represent the 25th to 75th percentiles, with the line within each box representing the mean values, and the whiskers identifying the minimum and maximum values.

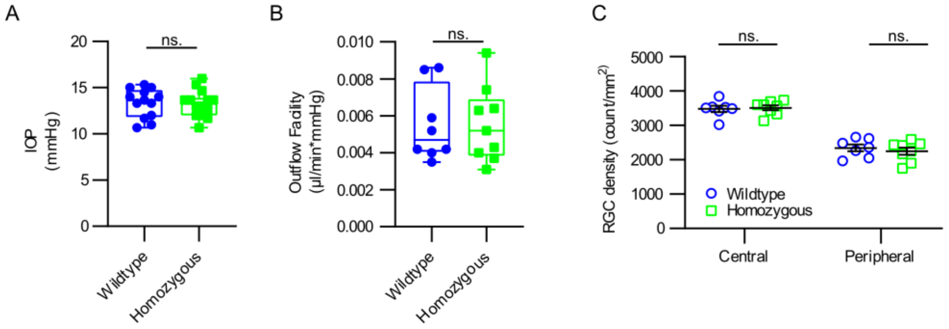


**Supplemental Figure 9. One month old *Thbs1*$^{R1034C}$ homozygous mutant mice do not show features of the glaucomatous phenotype**. At four weeks, compared to wild type mice the *Thbs1*$^{R1034C}$ mutants have similar (**A**) intraocular pressure (IOP) to wild type mice (N=13, 14, p=0.89, as determined by unpaired t-test) , (**B**) outflow facility (N=8, 9, p=0.98, as determined by unpaired t-test) (**B**), and (**C**) retinal ganglion cell (RGC) counts (N=7, 8, p=0.68, as determined by two-way ANOVA) to the wildtype controls. ns., not significant. The box boundaries represent the 25th to 75th percentiles, with the line within each box representing the mean values, and the whiskers identifying the minimum and maximum values. Data in panel C represent the means ± SEM.

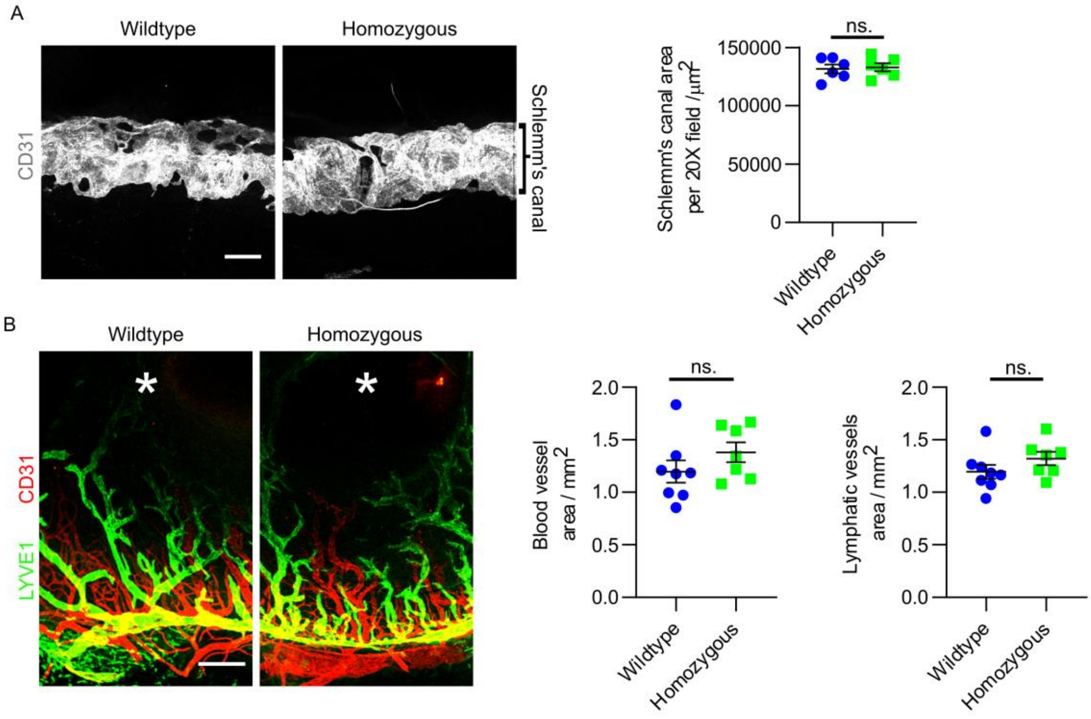


**Supplemental Figure 10. THBS1$^{R1034C}$ does not affect the Schlemm's canal development and corneal lymph- and angiogenesis.** (**A**) Staining of CD31 showed similar Schlemm's canal morphology between wildtype and homozygous mice at the age of 8 months. For each group, four 20X fields on four quadrants were captured per eye (n=6, p=0.82). Scale bars: 100 µm; 20X fields comprise an area of 0.501 mm$^2$. (**B**) There was no significant difference of lymph- (LYVE1 staining) or angiogenesis (CD31 staining) to bFGF pellet (20ng) between 4-month-old wildtype and homozygous mice. * indicates the location of pellet. The blood vessel and lymphatic vessel area were obtained from whole cornea (8 eyes from wildtype controls and 7 from homozygous, p=0.22 and 0.19, respectively). Scale bars: 200 µm. Data represent the means ± SEM. P-value was determined by Student's t test. ns., not significant.

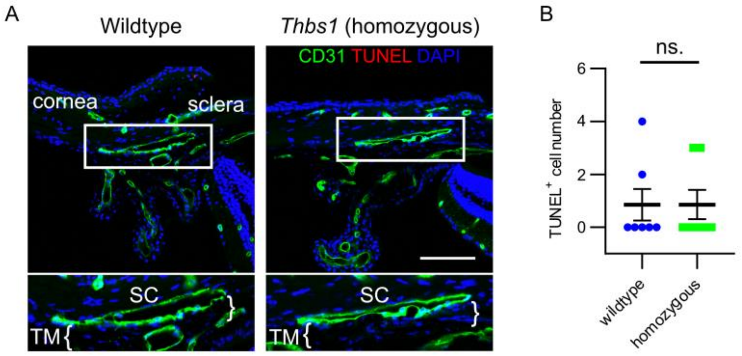


**Supplemental Figure 11. Cell death was not induced in *Thbs1* homozygous mutants**. In situ cell death was detected by TUNEL assay in 6 months old *Thbs1*$^{R1034C}$ homozygous and age-matched wildtype mice (**A**). Scale bar, 100 μm. TUNEL+ cells in trabecular meshwork (TM) were counted from 6 random sections of the eye. (**B**) There was no significant difference in the number of dead TM cells between mutants and wildtype controls. N=7, data represent the means ± SEM. p>0.99 as determined by unpaired t-test. ns., not significant.

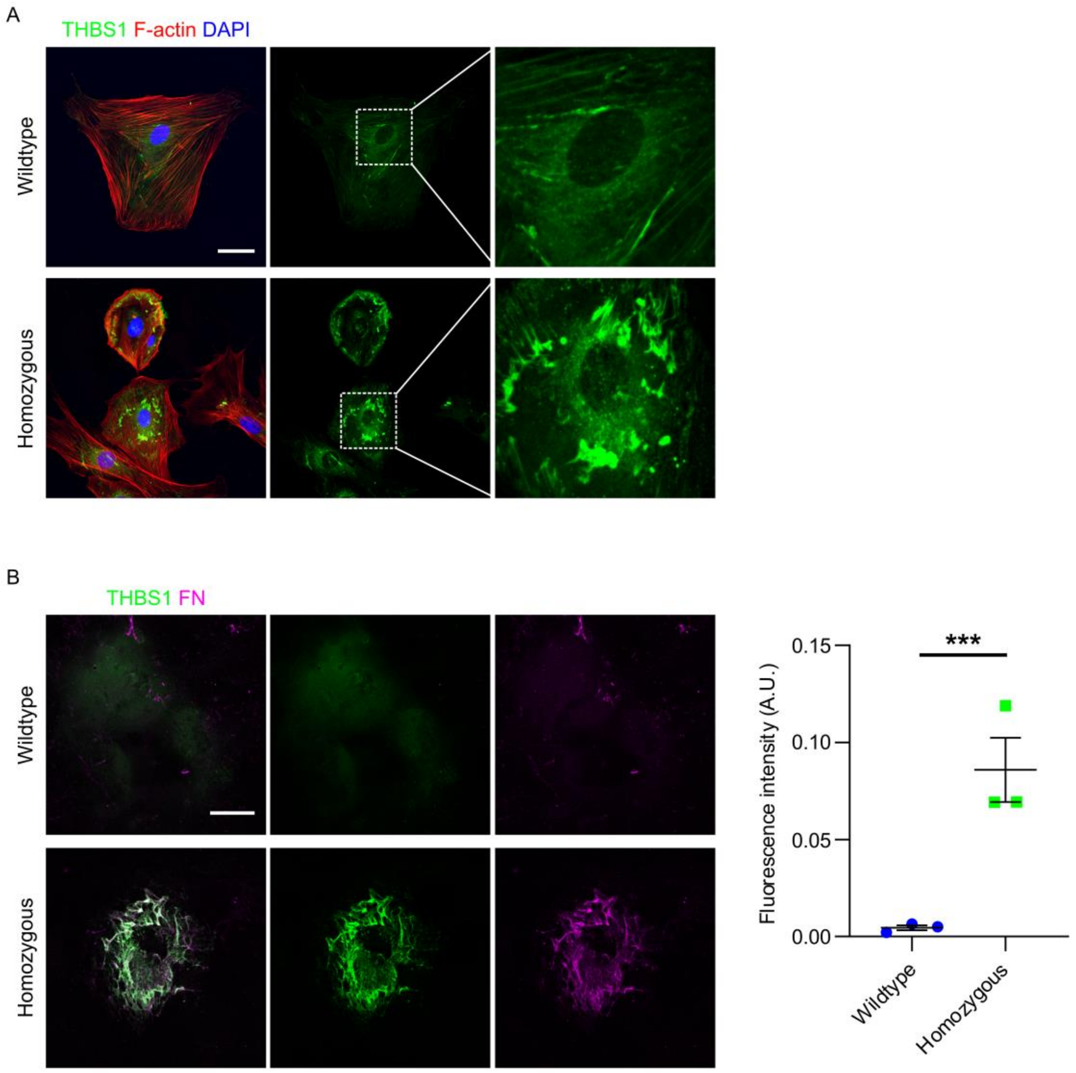


**Supplemental Figure 12. R1034C mutation promotes the ECM deposition of TM cell-derived THBS1.** Primary TM cells were isolated from both 2-month-old *Thbs1*$^{R1034C}$ homozygous and age-matched wildtype animals. (**A**) Aggregation of TM cell-derived THBS1 protein was evident in mutant cells. (**B**) To determine the location of the aggregates, cellular components were removed by $NH_4OH$, and the ECM was visualized through immunofluorescence staining. Compared to wildtype, mutant TM cells deposited significantly more THBS1 protein in the ECM (N=3). Notably, the mutated THBS1 protein was also co-localized with fibronectin (FN). Those findings corroborated the in vivo results from mice (Figure 4, 5 and 8). A.U., arbitrary fluorescence units. Data represent the means ± SEM. P-value was determined by Student's t test. *** p<0.0001. Scale bars: 50 μm.

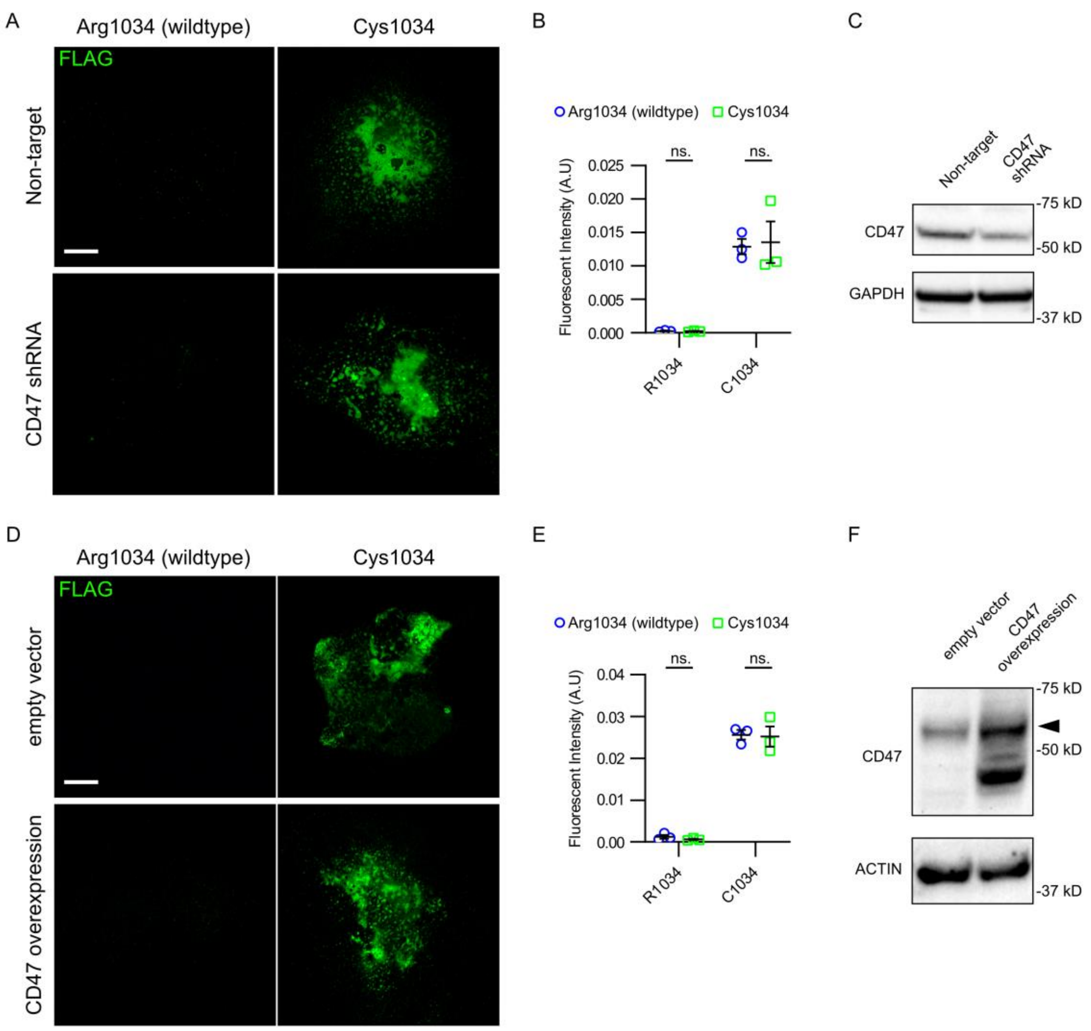


**Supplemental Figure 13. CD47 is not involved in the formation of extracellular THBS1$^{C1034}$ deposition.** A stable CD47 knockdown COS-7 cell line was generated by transducing CD47 shRNA using a lentiviral system. Compared to the non-targeting shRNA, the CD47 shRNA successfully reduced the expression of CD47 (**C**). Wildtype (THBS1$^{R1034}$) and mutant (THBS1$^{C1034}$) expressing plasmids (FLAG tagged) were then transfected to the non-targeting and CD47 shRNA cell lines, respectively. The THBS1 ECM deposition was quantified using anti-FLAG immunofluorescent staining. (**A, B**) Neither the wildtype nor the mutant THBS1 was affected by the reduction of CD47. N=3, data represent mean ± SEM. p=0.856 as determined two-way ANOVA. A stable CD47 overexpression COS-7 cell line was generated through transduction of CD47 expressing lentivirus. Empty vector was used as negative control. The overexpression was confirmed by western blot (**F**). Similarly, THBS1 expressing plasmids were transfected in the two cell lines. (**D, E**) Neither wildtype nor the mutant THBS1 was affected by the increased CD47 (black arrowhead). N=3, data represent mean ± SEM. p=0.714 as determined two-way ANOVA. Scale bar, 10 μm. ns., not significant.

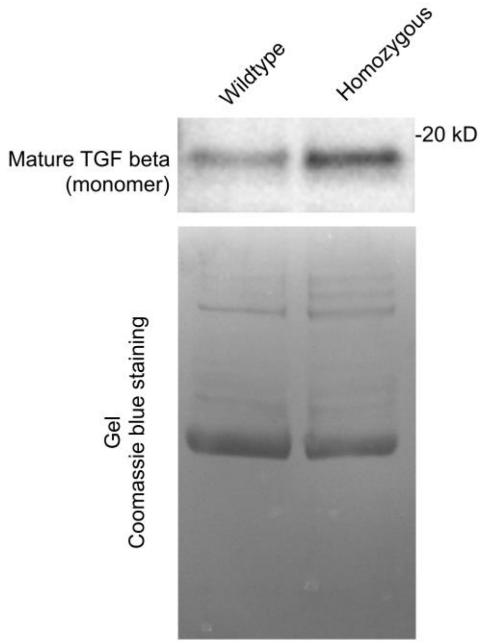


**Supplemental Figure 14. Increased TGF beta activation in the trabecular meshwork (TM) cells of *Thbs1*$^{R1034C}$ mutants compared to wildtype controls.** Western blot from primary TM cell (isolated from 2-month-old mice) conditioned media showed that more of the active form of TGF beta (size: 15kDa) was present in the mutant cell culture than the wildtype. Coomassie blue staining of SDS page gel showing below indicated even protein loading in the experiments.

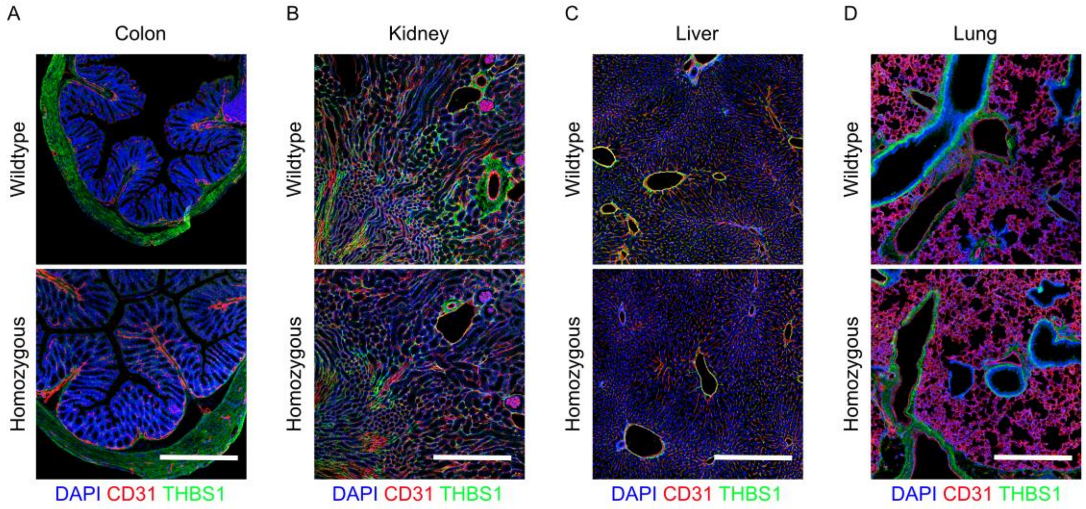


**Supplemental Figure 15. THBS1$^{R1034C}$ is not accumulated in colon, kidney, liver, and lung in mutant mice.** immunofluorescent staining of THBS1 (green) indicates comparable abundance of THBS1 in colon (**A**), kidney (**B**), liver (**C**), and lung (**D**) in the mutant and wild type mice at the age of 10 months. Blood vessels are co-stained by anti-CD31 antibody (red). Nuclei are labeled by DAPI (blue). Scale bar, 500 μm.

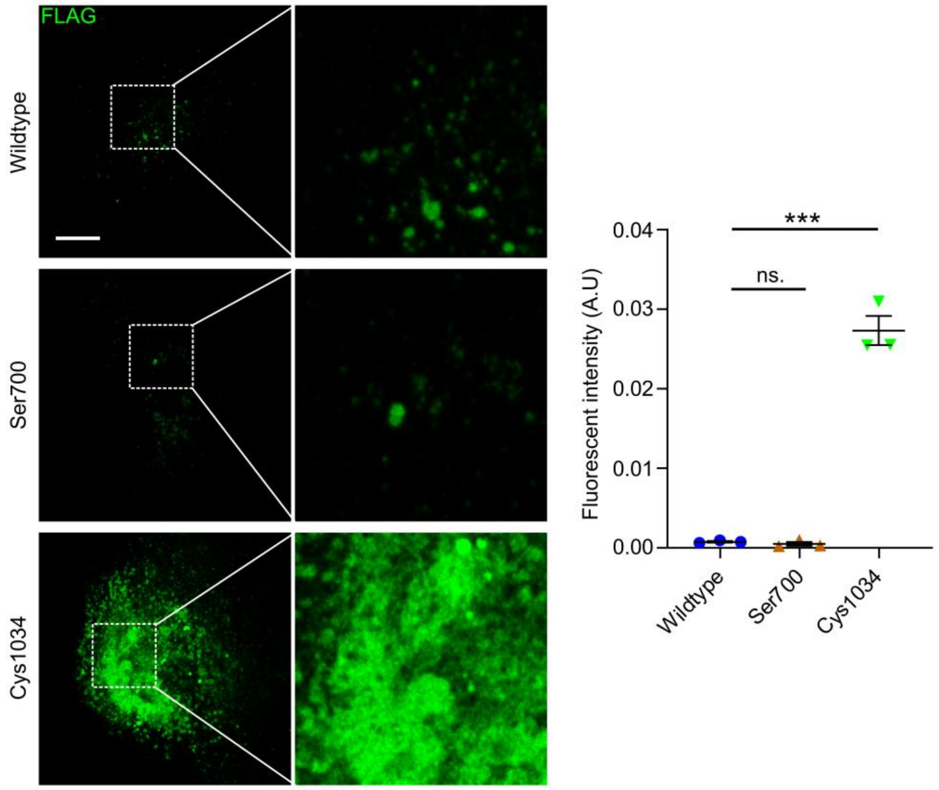


**Supplemental Figure 16. N700S does not promote THBS1 deposition in ECM.** THBS1 expression plasmids (FLAG-tagged) with mutation of R1034C, and N700S were transfected in COS-7 cells, respectively. After removal of cells, extracellular THBS1 was evaluated by FLAG immunofluorescence staining. There was significantly more deposition of $THBS1^{R1034C}$ compared to wildtype and $THBS1^{N700S}$. However, the amount of deposition between $THBS1^{N700S}$ and wildtype control was comparable. N=3, P-value was determined by one-way ANOVA followed by multiple comparisons. *** p<0.0001, ns., not significant. Scale bars: 10 μm. A.U., arbitrary fluorescence units. Data represents the mean ± SEM.
